# Evaluation and Validation of Assembling Corrected PacBio Long Reads for Microbial Genome Completion via Hybrid Approaches

**DOI:** 10.1371/journal.pone.0144305

**Published:** 2015-12-07

**Authors:** Hsin-Hung Lin, Yu-Chieh Liao

**Affiliations:** Institute of Population Health Sciences, National Health Research Institutes, Miaoli County, Taiwan; Universiteit Utrecht, NETHERLANDS

## Abstract

Despite the ever-increasing output of next-generation sequencing data along with developing assemblers, dozens to hundreds of gaps still exist in *de novo* microbial assemblies due to uneven coverage and large genomic repeats. Third-generation single-molecule, real-time (SMRT) sequencing technology avoids amplification artifacts and generates kilobase-long reads with the potential to complete microbial genome assembly. However, due to the low accuracy (~85%) of third-generation sequences, a considerable amount of long reads (>50X) are required for self-correction and for subsequent *de novo* assembly. Recently-developed hybrid approaches, using next-generation sequencing data and as few as 5X long reads, have been proposed to improve the completeness of microbial assembly. In this study we have evaluated the contemporary hybrid approaches and demonstrated that assembling corrected long reads (by runCA) produced the best assembly compared to long-read scaffolding (*e*.*g*., AHA, Cerulean and SSPACE-LongRead) and gap-filling (SPAdes). For generating corrected long reads, we further examined long-read correction tools, such as ECTools, LSC, LoRDEC, PBcR pipeline and proovread. We have demonstrated that three microbial genomes including *Escherichia coli* K12 MG1655, *Meiothermus ruber* DSM1279 and *Pdeobacter heparinus* DSM2366 were successfully hybrid assembled by runCA into near-perfect assemblies using ECTools-corrected long reads. In addition, we developed a tool, Patch, which implements corrected long reads and pre-assembled contigs as inputs, to enhance microbial genome assemblies. With the additional 20X long reads, short reads of *S*. *cerevisiae* W303 were hybrid assembled into 115 contigs using the verified strategy, ECTools + runCA. Patch was subsequently applied to upgrade the assembly to a 35-contig draft genome. Our evaluation of the hybrid approaches shows that assembling the ECTools-corrected long reads via runCA generates near complete microbial genomes, suggesting that genome assembly could benefit from re-analyzing the available hybrid datasets that were not assembled in an optimal fashion.

## Introduction

Determining microbial genomes is an essential prerequisite to understanding microbial biology. With the advent of next-generation sequencing (NGS) technology, sequencing microbial genomes is now affordable at decentralized laboratory scales and sequence information can be rapidly obtained. Compared to traditional Sanger sequencing technology, NGS technologies offer several distinct features, such as large read volumes and concise lengths. Such technological advances have dramatically improved in throughput and quality, and, in parallel with these improvements, numerous algorithms have been proposed for *de novo* sequence assembly [1]. However, many microbial genomes in the Genomes Online Database (GOLD) are incomplete, which reveals the limitation of NGS—long repeats present in multiple copies cannot be solved by means of using short reads [2–4].

Currently, third-generation single-molecule, real-time (SMRT) sequencing technology from Pacific Biosciences (PacBio) has been used to generate long reads, facilitating the assembly of complete microbial genomes [3, 5–8]. However, the error rates of single-molecule reads are high. Hybrid assembly methods, such as A Hybrid Assembler (AHA) [5] and PacBio corrected reads pipeline (PBcR pipeline) [6], were therefore proposed to avoid and address these limitations—via scaffolding using long read sequence information and by correcting the errors using short reads; however, some of the assemblies remain un-finished [9, 10]. Non-hybrid methods including hierarchical genome-assembly process (HGAP) and PBcR pipeline via self-correction have been proposed recently to complete microbial genome assemblies from long-read SMRT sequencing data [3, 8]. Based on the simulated PacBio reads investigated in Koren *et al*.'s publication [3], 150X is the recommended sequencing depth to maximize assembly continuity using C2 chemistry, which is equivalent to 8 SMRT cells for a 5 Mb genome using the RS sequencing system (> 100 Mb throughput per SMRT cell for the RS instrument). Currently, the PacBio RS II system can generate an increased number of longer reads (over 250 Mb throughput per SMRT cell), thus bacterial genomes have been successfully assembled *de novo* by the non-hybrid approach (HGAP or PBcR pipeline) using one or two SMRT cells [11–13]. However, because the non-hybrid approaches require high coverage (> 50X) [14], it can be prohibitively expensive for a relatively large microbial genome size [15]. Furthermore, it is a shame to discard short reads that were previously sequenced. As a result, hybrid methods that use long reads to scaffold short-read assembly have been adopted in upgrading bacterial genome assemblies; several of these include AHA [5], Cerulean [16] and SSPACE-LongRead [17]. SPAdes 3.0 is a hybrid assembler that takes short and long reads as inputs [10, 18, 19]; it uses long reads for gap closure and repeat resolution. The PBcR pipeline uses short reads to trim and correct the PacBio long reads followed by the *de novo* assembly of the PacBio corrected reads to generate consensus sequences [3]. Unlike PBcR pipeline, ECTools uses pre-assembled unitigs constructed from short reads for long-read correction, which allows its successful application towards eukaryotic genome assemblies (genome size <100 Mb) [15]. Although some of the hybrid approaches have been evaluated for bacterial genome assemblies [10, 12, 17], little is known about their performance on the eukaryotic genome. In addition, long-read correction tools have been recently developed to improve correction accuracy and efficiency, *e*.*g*., LoRDEC [20], LSC [21] and proovread [22]. However, the assembly performance of those corrected long reads remains unclear.

In this work, we compare the state-of-the-art hybrid approaches and developed a tool, named Patch, to exploit corrected long reads to enhance hybrid assemblies. We validated a hybrid approach (ECTools + runCA) to produce near-perfect assemblies for three bacterial species using additional 20X PacBio long reads. In addition, Patch along with the validated approach, was successfully employed to produce a genome assembly of yeast (an eukaryotic microorganism), which is represented by 35 contigs.

## Materials and Methods

### Materials

With respect to *Escherichia coli* K12 MG1655 (*E*. *coli*), 151-bp paired end library reads and one SMRT cell data (SMRT1) described in S. Koren *et al*.'s publication [3] were used in this study. An additional SMRT cell dataset of *E*. *coli* (SMRT2) provided in Pacific Biosciences’ DevNet (http://pacificbiosciences.github.io/DevNet/) was utilized. The Illumina Miseq data were assembled by Abyss 1.3.4 [23] and SPAdes 2.5 [18] separately. SMRT Analysis v2.0.1 was leveraged to filter subreads (continuous long reads) from the SMRT cells to obtain the filtered subreads of SMRT1 and SMRT2. Scaffolding tools, including AHA in SMRT Analysis v2.0.1 [5], Cerulean v0.1.1 [16] and SSPACE-LongRead v1-1 [17], were utilized in this study. SPAdes 3.0 was used for the hybrid assembly of short and long reads [18]. The subreads of SMRT1 (and SMRT2) were corrected with Miseq sequencing data to CPBLRs (corrected PacBio long reads) by invoking the PBcR command with the parameters: -length 500, -partitions 200 [24]; the PBcR pipeline (wgs-8.2) was used in this study. LoRDEC 0.4.1 [20] and LSC 0.3.1 [21] were also used separately to produce CPBLRs with default parameters. Additionally, the subreads were corrected by ECTools using Abyss-assembled unitigs [15]. In addition to the short reads, the unitigs were used for long-read correction by proovread 2.12 [22]. The CPBLRs were then assembled alone by Celera Assembler (runCA) [3]. Pre-assembled contigs and CPBLRs were input to Patch for upgrading genome assembly. All command-line references for data process and assembly are given in S1 Appendix. The resulting assemblies were evaluated with QUAST 2.3 [25] along with the reference genome (NCBI reference sequence NC000913.2) and gene list.

We further applied the long-read correction tools to two other bacterial species, *Meiothermus ruber* DSM1279 (NC_013946.1, *M*. *ruber*) and *Pedobacter heparinus* DSM2366 (NC_013061.1, *P*. *heparinus*). A single SMRT cell was downloaded for *M*. *rube*r and *P*. *heparinus*, respectively [8]. Additional NGS data deposited in the NCBI Sequence Read Archive (SRA), including 454 sequencing data of *M*. *Ruber* (SRR017780) and Illumina MiSeq data of *P*. *heparinus* (SRR812176), was used. Short reads were assembled with Abyss and Newbler for *P*. *heparinus* and *M*. *ruber*, respectively. The short reads or the pre-assembled unitigs (Abyss-assembled unitigs or Newbler-assembled contigs) were also utilized by the above-mentioned correction tools for CPBLRs generation. The CPBLRs were assembled *de novo* by runCA.

In addition to the three bacterial species, the hybrid approach was applied to assemble the genome of *S*. *cerevisiae* W303 (yeast), whose sequencing data including short reads (of a 300-bp paired end library) and long reads (of 16 RS II SMRT cells), both available for download: http://schatzlab.cshl.edu/data/ectools/ [15]. Abyss was used to assemble the Illumina short reads, long reads from a single SMRT cell (m131225_191238_42137) were subsequently corrected using ECTools with the Abyss-assembled unitigs. The corrected long reads (CPBLRs) were assembled *de novo* by runCA. Patch was applied separately to upgrade the Abyss-assembled contigs and runCA-assembled contigs using the ECTools-corrected long reads.

### Algorithms of Patch

Patch consists of three major steps: (1) assembly modification, (2) representative contig/CPBLR selection, and (3) iterative connection to improve genome assembly.

#### Assembly modification

**Fig 1:**
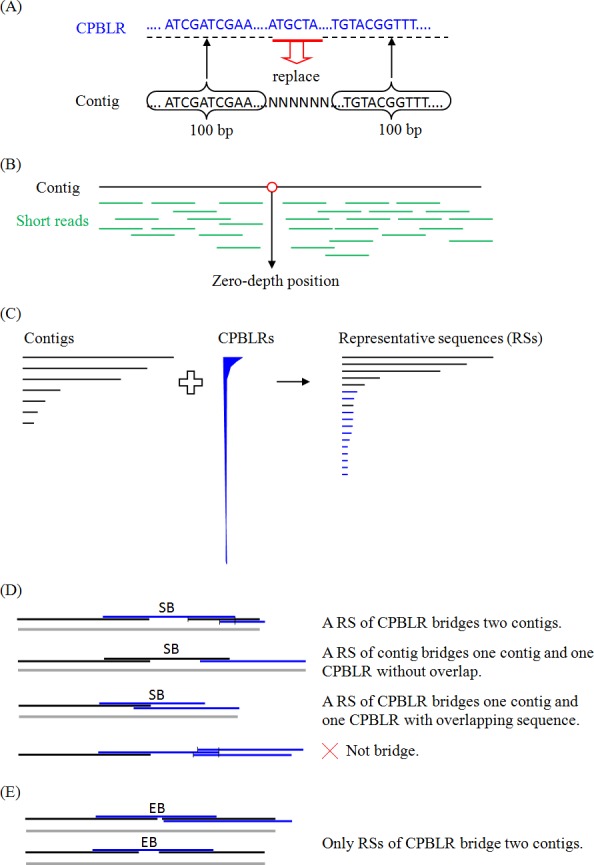
Schematic diagrams of the process flow used in Patch. (A) Gap-filling: a gap (with N’s) is replaced with the corresponding sequence (e.g., ATGCTA). (B) Splitting: A contig is split into two sequences at the zero-coverage position. (C) Representative sequence selection: representative sequences are selected from a modified assembly (contigs in black color) combined with all/15X CPBLRs (in blue color). (D) Strict bridging (SB): a representative sequence (RS) is used for bridging if it uniquely (*i*.*e*. the length of end-to-end alignments (as bounded by the vertical bars) is maximal and at least 1.25-fold larger than the secondary choice) aligns to two representative sequences (either one must be a black representative contig). The grey lines represent connected sequences. (E) Easy bridging (EB): a representative CPBLR (in blue) is used for bridging if it aligns to two individual contig sequences (in black) at either end.

As shown in Fig 1A, any N within a contig defines a gap. The 100-bp flanking sequences of the gap in the contig were used to identify the spanning-gap CPBLRs. The gap was replace with the corresponding sequence (*e*.*g*., ATGCTA) which has maximal occurrences (>2). Single-end or paired-end library reads were aligned to the gap-filled contigs by SOAP2 [26] to estimate read coverage statistics. As shown in Fig 1B, contigs were split at the zero-coverage positions except the regions located at the end of contigs (<1% contig length). Contigs split into segments smaller than 50 bp were discarded.

#### Representative contig/CPBLR selection

The longest 15X CPBLRs of the genome size (if a pre-defined genome size is available) or all CPBLRs were combined with the modified assembly for representative sequence selection (Fig 1C). NUCmer [27] was employed with the default setting to perform sequence alignment (one vs. all-minus-one). The longest sequence among contigs and CPBLRs was selected as a representative sequence; contigs and CPBLRs whose major portion of sequence (>95% alignment rate and sequence identity) was found in the representative sequence were removed. The longest sequence of the remaining sequences was iteratively selected as a subsequent representative sequence until no sequence remained.

#### Iterative connection

The representative sequences were self-aligned (all vs. all) by blastn to identify end-to-end overlaps. If a representative sequence uniquely bridged either two representative contigs or one representative contig and one CPBLR, the representative sequence was selected as a candidate for connection. Here, we defined "strict bridging" for the unique bridge whose ends were aligned to a single end of the sequence of choice, *i*.*e*. the length of end-to-end alignment was maximal and at least 1.25-fold larger than the secondary choice (Fig 1D). The candidate CPBLRs were ordered by length in descending order to bridge two adjacent sequences. If the two adjacent sequences bridged by a CPBLR overlapped, the overlapping sequence of the shorter segment was trimmed to connect with the other sequence. If a CPBLR spanned a gap between two adjacent sequences, the gap was filled with the corresponding CPBLR sequence. This process was performed iteratively until no CPBLR remained for bridging. We then employed the connected sequences (grey lines in Fig 1D) as candidates for connection and performed strict bridging iteratively. Once the sequences were ruled out for strict bridging, the connected sequences and the representative sequences that were not used for connection were self-aligned to estimate the maximal size of the repetitive regions. Subsequently, we examined each of the remaining representative CPBLRs to determine whether they were capable of bridging two sequences (not CPBLR). As shown in Fig 1E, both ends of a CPBLR (blue line) are end-to-end overlapping with only two individual contig sequences (black lines). Such a CPBLR was selected as a candidate for “easy bridging” if its length was greater than the maximum size of the repetitive regions. Prior to discarding the representative CPBLRs that were unable to bridge, the sequences delineated by these CPBLRs (in step 2, representative sequence selection) were examined for easy-bridging candidates. Subsequently, the candidate CPBLRs were ordered by length in a decreasing fashion to bridge two adjacent contigs. After the strict and easy bridging processes, the representative contigs and the connected sequences were self-aligned (all vs. all). If two sequences comprised a proper end-to-end overlap and the overlapping length was greater than the maximum size of the repetitive regions, they were connected. Finally, an alternative easy-bridging process was iteratively performed to ensure each CPBLR bridging candidate had been used. Details of the Patch algorithm are provided in S2 Appendix.

## Results

### Comparison of hybrid assembly strategies

**Fig 2:**
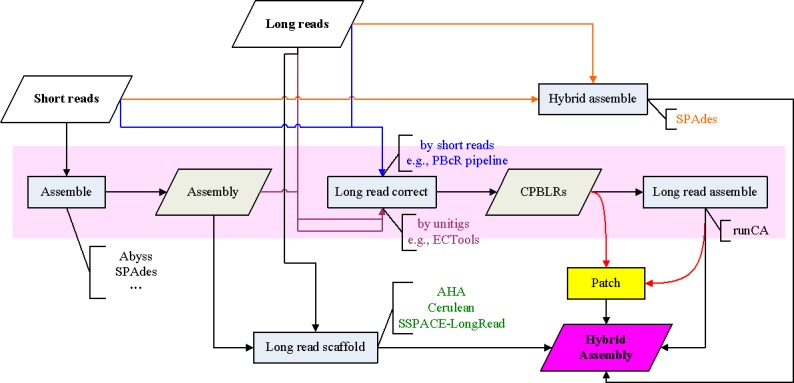
A workflow for producing hybrid assemblies. Top: SPAdes directly synthesizes short and long reads as inputs to generate hybrid assemblies. Middle: long reads are corrected by PBcR pipeline or by ECTools using short reads and pre-assembled unitigs, respectively, and then the corrected long reads (corrected PacBio long reads, CPBLRs) are assembled *de novo* by runCA. Bottom: AHA, Cerulean and SSPACE-LongRead are scaffolders that use long read information to scaffold pre-assembled contigs constructed from short reads. In this study, Patch is designed to enhance the hybrid assembly with the corrected long reads (CPBLRs).

**Fig 3:**
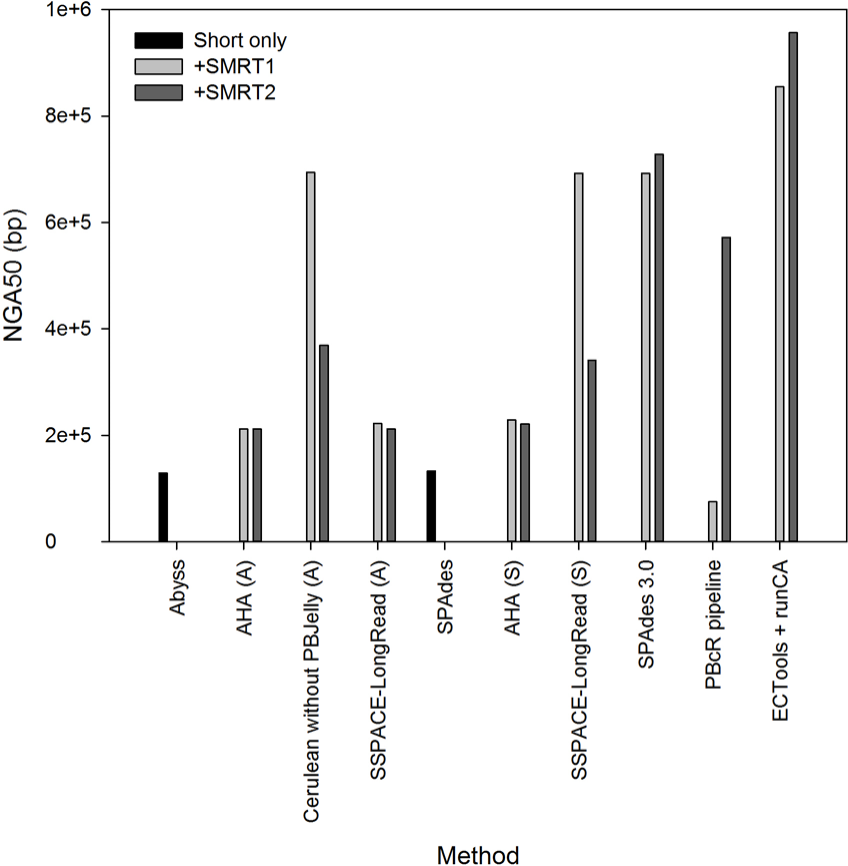
Hybrid assembly performance measured by NGA50 length. Abyss-assembled contigs (A) and SPAdes-assembled contigs (S) from short reads were separately scaffolded by the scaffolders including AHA, Cerulean and SSPACE-LongReads using long reads (SMRT1 and SMRT2). SPAdes 3.0 and PBcR pipeline synthesized the short and long reads as inputs. ECTools + runCA: the ECTools-corrected long reads were assembled *de novo* by runCA.

The contemporary approaches to produce hybrid assemblies using both short and long reads are summarized in Fig 2. As reported in the previous studies [10, 12], SPAdes produced hybrid assemblies by adding support for long reads. Short reads and pre-assembled unitigs (*e*.*g*. produced by Celera Assembler) were utilized by PBcR pipeline [6] and ECTools [15], respectively, for long read correction. The corrected PacBio long reads (CPBLRs) were then assembled by the Celera Assembler (runCA). AHA [5], Cerulean [16] and SSPACE-LongRead [17], are long-read scaffolders that are designed to scaffold pre-assembled contigs constructed by short reads using long reads. We performed these hybrid approaches on real data originating from *E*. *coli* and evaluated the as-produced hybrid assemblies with the reference genome by QUAST. The key metrics—the NGA50 length—of each assembly are shown in Fig 3. Please note that the NGA50 is the NG50 length by breaking contigs at misassembly events [25]; this metric is thereby an accurate and contiguous indicator for assembly evaluation. As can be seen in the figure, the NGA50 of the assemblies obtained from the long-read scaffolders, *e*.*g*. AHA, Cerulean and SSPACE-LongRead, are higher than that of the short-read assemblies produced by Abyss and SPAdes. However, the scaffolding performance, as given by the NGA50 length, depends on the nature of the data including the pre-assembled contigs (Abyss or SPAdes) and long reads (SMRT1 or SMRT2). For example, SSPACE-LongRead produced improved assemblies when scaffolding the SPAdes-assembled contigs than the Abyss-assembled contigs. This was also the case when using the long reads from SMRT1 when compared with those from SMRT2. In contrast, SPAdes 3.0 consistently produced optimal hybrid assemblies, given NGA50 lengths as high as 700 Kbp. In addition, the longest contigs of the SPAdes 3.0-produced hybrid assemblies exceeded 2.4 Mbp. As for assembling corrected long reads, the PBcR pipeline, using the Illumina short reads to correct the long reads, produced poor hybrid assemblies when using the insufficiently-long reads of SMRT1, both in length and amount (the average length and sequencing depth for SMRT1 are 2.0 Kbp and 16X; for SMRT2, 2.6 Kbp and 23X). Nevertheless, the assemblies produced by ECTools + runCA—the long reads were corrected by ECTools using the Abyss-assembled unitigs and subsequently assembled by runCA—exhibited the highest NGA50 lengths. Accordingly, as shown in Fig 3, we found that the ECTools-corrected long reads were assembled, *de novo*, into the most optimal assemblies among the approaches considered in this study.

### Validation of a hybrid approach (ECTools + runCA)

In the case of the hybrid assembly of the *E*. *col*i genome, we have shown that ECTools + runCA produced the most optimal assemblies among approaches examined, as shown in Fig 3. It is evident that long-read assembly outperformed long-read scaffolding. In this vein, long-read correction tools are worthy of further exploration. LoRDEC, LSC and PBcR pipeline are the correction tools for short reads, while ECTools synthesizes unitigs as the basis for long-read correction. Proovread allows unitigs to be used along with short reads for long-read correction. Please note that not every assembler produces unitigs. Among popular *de novo* assemblers for bacterial genome assembly [28], we are aware that SOAPdenovo2, SPAdes and Velvet do not produce unitigs. However, Abyss produced unitigs and was reported to be a suitable assembler with the most efficient CPU utilization [28]. We have input SOAPdenovo2-assembled and SPAdes-assembled contigs to ECTools for long read correction and subsequently assembled the corrected long reads. The assembly results were not as optimal when compared with the one using Abyss-assembled unitigs. We thus implemented Abyss for short-read assembly. As shown in the top of Table 1, the *E*. *coli* assembly produced by ECTools + runCA is near complete, with the largest contig of 4.6 Mbp. Although the assembly obtained from Proovread-corrected long reads gives a comparable NGA50 length of 949 Kbp to the ECTools assembly, the largest contig was only 3.5 Mbp. The other correction tools including LoRDEC, LSC and PBcR pipeline improved the accuracy of long reads so as to allow the corrected long reads to be assembled by runCA; nevertheless, these hybrid approaches were less efficient in producing contiguous assemblies. To further evaluate the hybrid approaches, we applied them to two other bacterial species, *P*. *heparinus* and *M*. *ruber*. It is worth noting that the shorts reads of *M*. *ruber* were generated from another NGS platform (Roach-454), and thus were assembled by Newbler. As detailed in Table 1, the QUAST-evaluated results consistently show that the hybrid approach, ECTools + runCA, produces near-perfect bacterial genome assemblies, with the largest contig near to the genome size. Diagonal-like dot plots of the sequence assemblies against the reference genomes (data not shown) also revealed the accuracy of assemblies produced by implementing the hybrid strategy. Although ECTools has been proposed and evaluated for *E coli* and several eukaryotic genome assemblies [15], this is the first study to demonstrate that ECTools-corrected long reads based on Abyss-assembled unitigs or Newbler-assembled contigs were successfully assembled by runCA into near-perfect assemblies, which were not obtained from the identical long reads corrected by other tools. In spite of the success of SPAdes 3.0 in assembling the *E coli* genome, it is worth noting that SPAdes 3.0 hybrid assembled the sequencing data of *P*. *heparinus*, but resulted in a hundred-contig assembly with the largest contig size equal to 1.8 Mbp. With the assessment of three bacterial genomes, the approach—ECTool + runCA—was corroborated to efficiently implement hybrid genome assembly.

**Table 1:** Comparison of hybrid assemblies obtained from various long-read correction tools.

Assembly	No. of contigs	Largest contig	Total length	NGA50	No. of misassemblies	No. of mismatches^a^	No. of indels^a^	Genome fraction (%)	No. of genes	Overall runtime
*Escherichia coli* K12 MG1655 (genome size = 4636675 bp, No. of genes = 4497)										
Abyss	82	285832	4646776	129004	2	2.92	0.41	99.48	4426	60m
LoRDEC + runCA	47	4188310	4791407	686189	43	3.30	0.65	100.00	4494	15h 46m
LSC + runCA	29	1954248	4769525	572337	21	2.26	0.54	100.00	4492	21h 35m
PBcR pipeline	12	1889858	4667990	572347	8	2.03	0.35	99.91	4484	4h 35m
ECTools + runCA	12	4644297	4695577	956540	14	9.33	1.21	99.99	4494	11h 21m
Proovread + runCA	6	3523306	4674021	949310	8	5.73	0.32	100.00	4494	5h 42m
*Pedobacter heparinus* DSM2366 (genome size = 5167383 bp, No. of genes = 4339)											
Abyss	44	1484728	5201989	403999	0	1.22	0.23	99.93	4309	90m
LoRDEC + runCA	30	1269137	5205089	792703	5	18.54	2.95	99.87	4329	2h 30m
PBcR pipeline	126	549480	5037466	73978	2	3.95	1.36	96.62	4075	15h 34m
ECTools + runCA	4	5164134	5177937	3895884	1	10.12	1.61	99.99	4335	8h 28m
Proovread + runCA	13	1561045	5181637	1269500	3	9.25	0.54	99.98	4329	12h 58m
*Meiothermus ruber* DSM1279 (genome size = 3097457 bp, No. of genes = 3105)											
Newbler	44	509970	3071504	136299	0	0.33	3.65	99.16	3046	10m
LoRDEC + runCA	25	1082032	3138975	646109	10	2.71	20.64	99.97	3099	2h 52m
PBcR pipeline	6	1927798	3096677	1927798	0	4.20	18.37	99.83	3093	23m
ECTools + runCA	1	3095849	3095849	3095849	0	1.29	3.97	99.95	3101	7h 7m
Proovread + runCA	65	295928	3089235	88185	2	2.40	7.45	99.64	3043	3h 47m

^a^ No. of mismatches/indels per 100 Kbp

### Improvement of hybrid assemblies using Patch

Patch was implemented in Python (S1 File, also available at http://sourceforge.net/projects/sb2nhri/files/Patch/) and it required pre-installation of BLAST+, MUMmer, and SOAP2. The inputs to Patch included a draft assembly and corrected PacBio long reads (CPBLRs); raw NGS reads were optional for assembly modification (split at the zero-coverage positions). Patch was applied to enhance the short-read pre-assembled contigs produced by Abyss using CPBLRs (corrected by the PBcR pipeline or ECTools), resulting in a higher average NGA50 of 450 Kbp than the one generated by long-read scaffolders (average NGA50 of 250 Kbp). The full QUAST-evaluated results are provided in S1 Table. As evident from Table 1, assembling corrected long reads could produce the largest contig close to the genome size, however, some assemblies contained more than one contig. The less-confident contigs (*i*.*e*. those with fewer reads) could be discarded from the runCA-produced assemblies with a cutoff value (*e*.*g*. 100 reads), as described in Koren *et al*.’s publication [3]; however this value depends on the quantity of long reads. Instead of establishing an arbitrary cutoff value, Patch was employed to isolate and remove redundant contigs from the assemblies produced by runCA. The twelve-contig and four-contig assemblies in Table 1 were reduced to two-contig assemblies for *E*. *coli* and *P*. *heparinus*, respectively. We concluded that for bacterial genome assembly, the hybrid approach, ECTools + runCA, produced near-perfect assemblies and the resulting assemblies still could be improved by implementing Patch.

Third-generation PacBio sequences have been used exclusively to reconstruct small microbial genomes [3, 8], however it can be relatively expensive to obtain the necessary coverage (> 50X) for large microbial genomes. In response to this issue, the hybrid strategy was utilized for the limited amount of long reads (~20X). Abyss was firstly conducted to generate a draft assembly of a yeast genome, which contains thousands of contigs (Table 2). The Abyss-assembled contigs were subsequently scaffolded by SSPACE-LongRead using the long read information. As shown in Table 2, SSPACE-LongRead could efficiently scaffold the assembly within 1.5 h, but it produced an exceptionally large genome size (over 19 Mbp) and a plethora of gaps (N's, over 20%) in the resulting assembly. By contrast, Patch was also applied to the Abyss-assembled contigs, and successfully produced the 81-contig yeast assembly. In comparison with the long-read scaffolding approach (*i*.*e*. SSPACE-LongRead), Patch provides an alternative to utilizing long reads for enhancing yeast genome assembly. SPAdes assembled the short and long reads for the yeast genome; however, it failed to execute properly on a high performance computing workstation (256 GB RAM) due to insufficient memory resources. The 579-contig assembly (in Table 2) was obtained from another workstation with 512 GB of RAM, nevertheless, this assembly had little superiority to the one generated by Abyss + Patch. We followed the verified hybrid strategy to correct the long reads to CPBLRs using ECTools with the Abyss-assembled unitigs. *De novo* assembly of the CPBLRs was subsequently performed via runCA. The runCA-assembled contigs contained erroneous duplications (resulting in a >13 Mbp genome size), nevertheless, Patch simultaneously removed the redundant sequences and increased the contiguity to the 35-contig assembly for yeast genome (see Figure A in S2 File for the accurate assembly).

**Table 2:** Comparisons of sequence assemblies for *S*. *cerevisiae* W303 with genome size of 12 Mbp.

Assembly	No. of contigs	Largest contig	Total Length	NGA50	# mismatches /100 Kbp	# N's /100 Kbp	Genome fraction (%)	Overall runtime
15 Gbp short reads and 235.7 Mbp long reads								
Abyss	2782	223330	13708216	77375	16.92	1.42	96.28	9h
Abyss + SSPACE-LongRead	2266	928510	19684054	177773	17.58	21237	96.38	10h 21m
**Abyss + Patch**	**81**	**579823**	**12298533**	**160249**	**17.68**	**0.02**	**95.66**	**67h** ^a^
SPAdes	579	745904	12205747	232200	22.13	0	96.20	90h^b^
ECTools + runCA	115	889557	13221295	198391	19.55	0	96.05	69h 45m^a^
runCA + SSPACE-longRead	101	1529179	13227377	228460	20.98	45.98	96.05	70h 16m
**ECTools + runCA + Patch**	**35**	**1528116**	**12203626**	**237082**	**19.17**	**0**	**95.82**	**70h 35m** ^a^

^a^ ECTools was used to correct the PacBio long reads using the Abyss-assembled unitigs, which took the runtime of 47 h.

^b^ We performed all analysis on a server (Intel Xeon E7-4820, 2.00GHz with 256 GB of RAM). However, SPAdes crashed on this server, this assembly was thus computed on another sever with 512 GB of RAM.

## Discussion

### Applications of hybrid approach

Next-generation sequencing technologies have significant limitations, especially short read lengths and amplification biases, which invariably leads to fragmented assemblies. Currently, a new technology—SMRT sequencing from PacBio—has been developed to generate kilobase-long reads. Therefore, it has become practical to complete bacterial genomes using the proposed approaches [3, 8]. Unfortunately, those approaches recommended 150X sequencing depth for the *de novo* assembly of a complete genome, which approximately requires 6–8 SMRT cells produced by the PacBio RS I system for a bacterium. Because a substantial body of NGS data has already been produced and employed to assemble draft genomes, the sequencing of an additional 8 SMRT cells represents an economic burden; here we examined the hybrid approach to upgrade draft genome assemblies with single SMRT cell data (~20X). We validated that the hybrid approach (ECTools + runCA) successfully produced near-perfect bacterial genome assemblies, including *E*.*coli*, *P*. *heparinus* and *M*. *ruber*, to include more than 99.9% encoded genes therein. We therefore recommend that the available hybrid datasets be re-analyzed by executing the verified hybrid strategy. The current instrument, the PacBio RS II system, can produce a greater number of longer reads (read lengths >20 Kbp and over 500 Mbp throughput per SMRT cell), therefore, it is becoming practical to complete small microbial genomes via the non-hybrid approach using RS II data (of a couple of SMRT cells) [11, 12]. Moreover, the MinHash Alignment Process (MHAP) was introduced (in wgs-8.3) to improve the computational efficiency and assembly performance associated with processing large genomes [13]. However, we have determined that the limited amount of long reads (~20X) associated with the three bacterial species listed in Table 1 were not assembled optimally by wgs-8.3 (see S2 Table for QUAST-evaluated results). Therefore, it still remains an economic burden to obtain the required amount of sequences when aiming to assemble a large microbial genome. We thus validated the approach (ECTools + runCA) to hybrid assemble yeast genome with limited long reads. We subsequently applied Patch to improve the hybrid assembly. As evident in Table 2, the previously proposed tools including SSPACE-LongRead and SPAdes did not perform well in the hybrid assembly of the yeast genome. By contrast, Patch, along with the hybrid approach (ECTools + runCA), indeed produced a high-quality draft assembly of the yeast genome. Please note that the most recent single molecule, real-time sequencer, the Sequel System, was unveiled on Sep. 30, 2015. The system delivers about 7X more reads than the RS II system, which hence poises the Sequel System for extensive use in future genome assemblies.

### Limitations of long-read scaffolding

To utilize PacBio long reads in order to improve genome assemblies, we have attempted to employ scaffolders including AHA, Cerulean and SSPACE-LongRead on the data of *E*. *coli*, but were unable to obtain satisfactory results. Although AHA and SSPACE-LongRead scaffolded the pre-assembled contigs with the support of PacBio long reads to reduce the number of contigs, they both produced assemblies with many unknown nucleotides (over 100 N's per 100 Kbp), as shown in S1 Table, which may impede gene characterization. For addressing such gaps, we have employed a gap-closing tool, PBJelly [29], to post-process the assemblies scaffolded by AHA and SSPACE-LongRead with the PacBio reads. PBJelly was applied to the AHA-scaffolded assembly with SMRT1 data, hence the number of N's per 100 Kbp was appreciably reduced from 382.11 to 108.55 and the number of genes was increased from 4426 to 4445 (S1 Table). However, the execution of PBJelly resulted in numerous misassemblies (Figure B in S2 File); a similar result was observed in applying PBJelly to the SSPACE-LongRead-scaffolded assembly (Figure C in S2 File). On the other hand, PBJelly was implemented in Cerulean for post-processing. Cerulean (including PBJelly) scaffolded the 82 Abyss-assembled contigs into the 24-contigs assembly using the SMRT1 long reads but produced 50 misassemblies (S1 Table). We therefore examined the outputs of Cerulean with and without PBJelly and found that substantial missassemblies were derived from PBJelly rather than Cerulean (Figure D in S2 File). In spite of this, the assemblies generated by Cerulean without PBJelly possessed an unsatisfactory total length of assemblies (exceptionally large genome size, >4.8 Mbp). This study tends to refute the benefits of long-read scaffolders, and suggests that long-read scaffolding may not be a practical way for upgrading genome assembly.

### Pros and cons of Patch

An intuitional bridge concept: if a single long read was aligned to pre-assembled contigs with end-to-end alignments, this long read is selected as a candidate for bridging (easy-bridging in Patch); a similar concept was adopted in PBJelly for gap-filling. However, this approach has been found to have two substantial issues: its inability to cross large gaps and its tendency to misjoin contigs (PBJelly misassemblies: Figure B-D in S2 File). We therefore developed Patch with three major steps in mind and focused our attention on the third step to address the above-mentioned problems. With PacBio long reads from a single SMRT cell, we have demonstrated that Patch indeed is able to enhance genome assemblies, especially when compared with long-read scaffolders; this is substantiated in S1 Table. In addition, Patch improved the yeast assembly produced by runCA to contain as few as 35 contigs and created a contig greater than 1.5 Mbp, as shown in Table 2. Patch did upgrade microbial genome assembly in terms of several assembly metrics, *e*.*g*. number of contigs, NGA50 and N’s. However, with the introduction of long reads, it increased minor errors (mismatches and indels in some cases). For example, to patch the assembly generated by Abyss using the ECTools-corrected reads (S1 Table), the numbers of mismatches and indels per 100 Kbp increased from 2.92 and 0.41 to 7.48–9.68 and 8.51–8.61, respectively. Similar results were observed when the ECTools-corrected long reads were assembled *de novo* by runCA (at the top and the middle of Table 1). Therefore, we have recognized that the CPBLRs produced by ECTools were not as accurate as short reads; nevertheless, the errors were as low as 0.01%.

## Conclusions

In summary, we have evaluated the hybrid approach and validated that the most optimal hybrid assemblies for microbial genomes were realized by correcting PacBio long reads using ECTools and subsequently assembling, *de novo*, the corrected long reads with runCA. We have also developed a software tool (Patch) that implemented corrected long reads and pre-assembled contigs along with optional short reads as inputs to improve microbial genome assemblies. Instead of sequencing high-coverage long reads, this study provides a validated hybrid strategy to produce high-quality microbial genome assemblies using short reads and long reads (~20X).

## Supporting Information

S1 Appendix Commands conducted for all experiments. (PDF)

S2 Appendix Details of Patch algorithm. (PDF)

S1 File Scripts of Patch algorithm. (ZIP)

S2 File Dot plots of sequence assembly against reference genome. Sequence assembly produced by Patch for *S*. *cerevisiae* W303 (Figure A). Sequence assemblies produced by AHA (Figure B), SSPACE-LongRead (Figure C) and Cerulean (Figure D) for *E*. *coli* MG1655.
(PDF)

S1 Table Quast-evaluated comparisons of hybrid assemblies. (XLS)

S2 Table Quast-evaluated comparisons of non-hybrid assemblies produced by PBcR pipeline in wgs-8.3. (XLS)

## References

[1] Nagarajan N, Pop M. Sequence assembly demystified. Nature reviews Genetics. 2013;14(3):157–67. Epub 2013/01/30. 10.1038/nrg3367 . 23358380

[2] Ferrarini M, Moretto M, Ward JA, Surbanovski N, Stevanovic V, Giongo L, et al An evaluation of the PacBio RS platform for sequencing and de novo assembly of a chloroplast genome. BMC Genomics. 2013;14(1):670 10.1186/1471-2164-14-670 24083400 PMC3853357

[3] Koren S, Harhay GP, Smith TP, Bono JL, Harhay DM, McVey SD, et al Reducing assembly complexity of microbial genomes with single-molecule sequencing. Genome Biol. 2013;14(9):R101 Epub 2013/09/17. 10.1186/gb-2013-14-9-r101 . 24034426 PMC4053942

[4] Huddleston J, Ranade S, Malig M, Antonacci F, Chaisson M, Hon L, et al Reconstructing complex regions of genomes using long-read sequencing technology. Genome Res. 2014 Epub 2014/01/15. 10.1101/gr.168450.113 . 24418700 PMC3975067

[5] Bashir A, Klammer AA, Robins WP, Chin CS, Webster D, Paxinos E, et al A hybrid approach for the automated finishing of bacterial genomes. Nature biotechnology. 2012 Epub 2012/07/04. 10.1038/nbt.2288 . 22750883 PMC3731737

[6] Koren S, Schatz MC, Walenz BP, Martin J, Howard JT, Ganapathy G, et al Hybrid error correction and de novo assembly of single-molecule sequencing reads. Nature biotechnology. 2012 Epub 2012/07/04. 10.1038/nbt.2280 . 22750884 PMC3707490

[7] Ribeiro FJ, Przybylski D, Yin S, Sharpe T, Gnerre S, Abouelleil A, et al Finished bacterial genomes from shotgun sequence data. Genome Res. 2012;22(11):2270–7. Epub 2012/07/26. 10.1101/gr.141515.112 22829535 PMC3483556

[8] Chin CS, Alexander DH, Marks P, Klammer AA, Drake J, Heiner C, et al Nonhybrid, finished microbial genome assemblies from long-read SMRT sequencing data. Nat Methods. 2013;10(6):563–9. Epub 2013/05/07. 10.1038/nmeth.2474 . 23644548

[9] Powers J, Weigman V, Shu J, Pufky J, Cox D, Hurban P. Efficient and accurate whole genome assembly and methylome profiling of E. coli. BMC Genomics. 2013;14(1):675 10.1186/1471-2164-14-675 24090403 PMC4046830

[10] Utturkar SM, Klingeman DM, Land ML, Schadt CW, Doktycz MJ, Pelletier DA, et al Evaluation and validation of de novo and hybrid assembly techniques to derive high quality genome sequences. Bioinformatics. 2014 10.1093/bioinformatics/btu391 . 24930142 PMC4173024

[11] Brown SD, Nagaraju S, Utturkar S, De Tissera S, Segovia S, Mitchell W, et al Comparison of single-molecule sequencing and hybrid approaches for finishing the genome of Clostridium autoethanogenum and analysis of CRISPR systems in industrial relevant Clostridia. Biotechnology for biofuels. 2014;7(1):40 10.1186/1754-6834-7-40 . 24655715 PMC4022347

[12] Liao YC, Lin SH, Lin HH. Completing bacterial genome assemblies: strategy and performance comparisons. Scientific reports. 2015;5:8747 10.1038/srep08747 25735824 PMC4348652

[13] Berlin K, Koren S, Chin CS, Drake JP, Landolin JM, Phillippy AM. Assembling large genomes with single-molecule sequencing and locality-sensitive hashing. Nature biotechnology. 2015;33(6):623–30. 10.1038/nbt.3238 . 26006009

[14] Koren S, Phillippy AM. One chromosome, one contig: complete microbial genomes from long-read sequencing and assembly. Current opinion in microbiology. 2014;23C:110–20. 10.1016/j.mib.2014.11.014 . 25461581

[15] Lee H, Gurtowski J, Yoo S, Marcus S, McCombie WR, Schatz M. Error correction and assembly complexity of single molecule sequencing reads. BioRxiv. 2014 10.1101/006395

[16] Deshpande V, Fung ED, Pham S, Bafna V. Cerulean: A hybrid assembly using high throughput short and long reads Algorithms in Bioinformatics: Springer; 2013 p. 349–63.

[17] Boetzer M, Pirovano W. SSPACE-LongRead: scaffolding bacterial draft genomes using long read sequence information. BMC Bioinformatics. 2014;15:211 10.1186/1471-2105-15-211 24950923 PMC4076250

[18] Bankevich A, Nurk S, Antipov D, Gurevich AA, Dvorkin M, Kulikov AS, et al SPAdes: a new genome assembly algorithm and its applications to single-cell sequencing. Journal of computational biology: a journal of computational molecular cell biology. 2012;19(5):455–77. Epub 2012/04/18. 10.1089/cmb.2012.0021 22506599 PMC3342519

[19] Prjibelski AD, Vasilinetc I, Bankevich A, Gurevich A, Krivosheeva T, Nurk S, et al ExSPAnder: a universal repeat resolver for DNA fragment assembly. Bioinformatics. 2014;30(12):i293–i301. 10.1093/bioinformatics/btu266 24931996 PMC4058921

[20] Salmela L, Rivals E. LoRDEC: accurate and efficient long read error correction. Bioinformatics. 2014;30(24):3506–14. 10.1093/bioinformatics/btu538 25165095 PMC4253826

[21] Au KF, Underwood JG, Lee L, Wong WH. Improving PacBio long read accuracy by short read alignment. PLoS One. 2012;7(10):e46679 Epub 2012/10/12. 10.1371/journal.pone.0046679 23056399 PMC3464235

[22] Hackl T, Hedrich R, Schultz J, Forster F. proovread: large-scale high-accuracy PacBio correction through iterative short read consensus. Bioinformatics. 2014;30(21):3004–11. 10.1093/bioinformatics/btu392 . 25015988 PMC4609002

[23] Simpson JT, Wong K, Jackman SD, Schein JE, Jones SJM, Birol I. ABySS: A parallel assembler for short read sequence data. Genome Research. 2009;19(6):1117–23. 10.1101/gr.089532.108 19251739 PMC2694472

[24] Junemann S, Sedlazeck FJ, Prior K, Albersmeier A, John U, Kalinowski J, et al Updating benchtop sequencing performance comparison. Nature biotechnology. 2013;31(4):294–6. Epub 2013/04/09. 10.1038/nbt.2522 . 23563421

[25] Gurevich A, Saveliev V, Vyahhi N, Tesler G. QUAST: quality assessment tool for genome assemblies. Bioinformatics. 2013;29(8):1072–5. Epub 2013/02/21. 10.1093/bioinformatics/btt086 23422339 PMC3624806

[26] Li R, Yu C, Li Y, Lam TW, Yiu SM, Kristiansen K, et al SOAP2: an improved ultrafast tool for short read alignment. Bioinformatics. 2009;25(15):1966–7. Epub 2009/06/06. 10.1093/bioinformatics/btp336 . 19497933

[27] Kurtz S, Phillippy A, Delcher AL, Smoot M, Shumway M, Antonescu C, et al Versatile and open software for comparing large genomes. Genome Biol. 2004;5(2):R12 Epub 2004/02/05. 10.1186/gb-2004-5-2-r12 14759262 PMC395750

[28] Junemann S, Prior K, Albersmeier A, Albaum S, Kalinowski J, Goesmann A, et al GABenchToB: A Genome Assembly Benchmark Tuned on Bacteria and Benchtop Sequencers. PLoS One. 2014;9(9):e107014 10.1371/journal.pone.0107014 25198770 PMC4157817

[29] English AC, Richards S, Han Y, Wang M, Vee V, Qu J, et al Mind the gap: upgrading genomes with Pacific Biosciences RS long-read sequencing technology. PLoS One. 2012;7(11):e47768 Epub 2012/11/28. 10.1371/journal.pone.0047768 23185243 PMC3504050

