# Supplementary material for: Evaluation and Validation of Assembling Corrected PacBio Long Reads for Microbial Genome Completion via Hybrid Approaches
Source: PLoS One. 2015 Dec 7;10(12):e0144305. doi: 10.1371/journal.pone.0144305 (PMC4671558; doi:10.1371/journal.pone.0144305)
Supplement: S1 Appendix — (PDF) [file pone.0144305.s001.pdf]

S1 Appendix

With respect to Escherichia coli K12 MG1655 (*E. coli*), we used the 151-bp paired end library reads and one SMRT cell data described in S. Koren *et al.*'s publication [1]. We further downloaded another SMRT cell dataset of *E. coli* provided in Pacific Biosciences' DevNet (http://pacificbiosciences.github.io/DevNet/).

MiSeq: Paired reads of *E. coli* are available at Illumina website. Mate1 and Mate 2 were downloaded separately.

Mate1:
ftp://webdata:webdata@ussd-ftp.illumina.com/Data/SequencingRuns/MG1655/MiSeq_Ecoli_MG1655_110721_PF_R1.fastq.gz

Mate2:
ftp://webdata:webdata@ussd-ftp.illumina.com/Data/SequencingRuns/MG1655/MiSeq_Ecoli_MG1655_110721_PF_R2.fastq.gz

Read length: 151bp

Read amount: 5,729,470 X2

Insert size ~ 300bp

We assembled the short reads using Abyss 1.3.4 [2]:

```
abyss-pe k=97 name=Abyss in='MiSeq_Ecoli_MG1655_110721_PF_R1.fastq
MiSeq_Ecoli_MG1655_110721_PF_R2.fastq'
```

SMRT1: Although the PacBio sequence reads are available at SRA (http://www.ncbi.nlm.nih.gov/sra/SRX255228), we cannot handle adapters correctly by using fastq-dump. We therefore requested for the h5 files from NCBI help desk. Files are listed below:

m120208_071634_42139_c100288480630000001523009507231245_s1_p0.bas.h5 (1.2GB)
m120208_122534_42139_c100290260310000001523009507231262_s1_p0.bas.h5 (1010MB)
m120208_160812_42139_c100290260310000001523009507231264_s1_p0.bas.h5 (733MB)
m120228_082105_42139_c100301722550000001523012308061200_s1_p0.bas.h5 (1.2GB)
m120228_100807_42139_c100301722550000001523012308061201_s1_p0.bas.h5 (1.0GB)
m120228_115504_42139_c100301722550000001523012308061202_s1_p0.bas.h5 (1.0GB)
m120228_134222_42139_c100301722550000001523012308061203_s1_p0.bas.h5 (985MB)
m120228_152936_42139_c100301722550000001523012308061204_s1_p0.bas.h5 (1.1GB)
m120228_171636_42139_c100301722550000001523012308061205_s1_p0.bas.h5 (1.1GB)
m120228_190630_42139_c100301722550000001523012308061206_s1_p0.bas.h5 (984MB)
m120228_192221_42129_c100298890010000001523009207231260_s1_p0.bas.h5 (1.1GB)

m120228_205404_42139_c100301722550000001523012308061207_s1_p0.bas.h5 (879MB)
m120228_210845_42129_c000304152550000001500000112311370_s1_p0.bas.h5 (1.2GB)
m120228_223624_richard_c001202352550000001500000112311330_s1_p0.bas.h5 (833MB)
m120229_004752_42129_c000304192550000001500000112311350_s1_p0.bas.h5 (936MB)
m120229_012852_42139_c000301732550000001500000112311360_s1_p0.bas.h5 (1.0GB)
m120229_193409_42129_c000304212550000001500000112311380_s1_p0.bas.h5 (1000MB)

We arbitrarily chose the first single SMRT cell (m120208_071634, corresponds to SRR797943) to run smrtpipe.py (SMRT analysis) with the following params.xml for getting the filtered subreads (*i.e.* continuous long reads).

```
<param name="minLength">
  <value>50</value>
</param>
<param name="readScore">
  <value>0.75</value>
</param>
<param name="minSubReadLength">
  <value>50< /value>
```

Statistics of the filtered subreads (SMRT1):

seqs amount:37077
seq avg len:2023.338161
total:75.02 Mb
depth: 16.13X

<u>SMRT2</u>: The h5 file was downloaded and unzipped from <u>http://files.pacb.com/datasets/primary-analysis/e-coli-k12/1.3.0/e-coli-k12-mg1655-raw-reads-1.3.0.tgz</u>. Similarly, we have run smrtpipe.py to get the filtered subreads.

Statistics of the filtered subreads (SMRT2):

seqs amount:41312
seq avg len:2584.021471
total:106.75 Mb
depth: 22.96X

<u>CPBLR1a & CPBLR2a</u>: The filtered subreads (SMRT1 and SMRT2) were corrected to long reads (corrected PacBio long reads, CPBLRs) via invoking the PBcR command (refer to <u>PBcR</u> for details, we have downloaded the version of 8.2

beta) along with the Miseq data:

fastqToCA -libraryname Miseq -insertsize 297 35 -mates MiSeq_Ecoli_MG1655_110721_PF_R1.fastq,MiSeq_Ecoli_MG1655_110721_PF_R2.fastq > MiSeqPE.frg

PBcR -length 500 -partitions 200 -l Pacbio_Illumina -s pacbio.spec -fastq subreads.fastq genomeSize=4650000 MiSeqPE.frg

Statistics of CPBLR1a:

seqs amount: 9993

seq avg len: 2981.43

total: 29.79 Mb

depth: 6.41X

Statistics of CPBLR2a:

seqs amount: 12123

seq avg len: 3624.92

total:43.94 Mb

depth: 9.45X

CPBLR1b & CPBLR2b: The filtered subreads (SMRT1 and SMRT2) were corrected to long reads (CPBLRs) by using ECTools (refer to ECTools for details) along with the Abyss-assembled unitigs.

Statistics of CPBLR1b:

seqs amount:22583

seq avg len:2626.54439

total:59.32 Mb

depth: 12.76X

Statistics of CPBLR2b:

seqs amount:33259

seq avg len:2611.3382

total:86.85 Mb

depth:18.68X

CPBLR2c: We also used LSC 0.3.1 [3] to correct the filtered subreads (SMRT2) using the short reads. However, it took the long runtime of 19 hr in correcting long reads and the accuracy of CPBLRs were not as good as the long reads corrected by

PBcR pipeline (see S4 Table for details).

Statistics of CPBLR2c:

seqs amount:29717

seq avg len:2813.7527

total:83.62 Mb

depth:17.98X

CPBLR2d: We used LoRDEC 0.4.1 [4] to correct the filtered subreads (SMRT2) using the short reads. It took the very short runtime of 7 min in correcting long reads:

lordec-correct -2 MiSeq_Ecoli_MG1655_110721_PF_R1.fastq,MiSeq_Ecoli_MG1655_110721_PF_R2.fastq -k 19 -s 3 -i subreads.fastq -o my-corrected-pacbio-reads-k19.fa

lordec-trim-split -i my-corrected-pacbio-reads-k19.fa -o my-corrected-pacbio-reads-k19-ts.fa

Statistics of CPBLR2d:

seqs amount:52470

seq avg len:1849.9352

total:97.07 Mb

depth:20.87X

CPBLR2e&f: We used proovread 2.12 [5] to correct the filtered subreads (SMRT2) using the short reads and the Abyss-assembled unitigs along with the short reads. It took around two hours in correcting long reads:

SeqChunker -s 60M -o SMRT2-%03d.fastq subreads.fastq

SMRT2-001.fastq ~ SMRT2-004.fastq

proovread -l SMRT2-001.fastq -s MiSeq_Ecoli_MG1655_110721_PF_R1.fastq -s MiSeq_Ecoli_MG1655_110721_PF_R2.fastq --pre SMRT2-001.cor

….

Statistics of CPBLR2e:

seqs amount:28712

seq avg len:2712.9368

total:77.89 Mb

depth:16.75X

SeqChunker -s 60M -o SMRT2-%03d.fastq subreads.fastq

SMRT2-001.fastq ~ SMRT2-004.fastq

proovread -l SMRT2-001.fastq -s MiSeq_Ecoli_MG1655_110721_PF_R1.fastq -s

MiSeq_Ecoli_MG1655_110721_PF_R2.fastq -u Abyss.utg.fa --pre SMRT2-001.cor --coverage 50

….

Statistics of CPBLR2f:

seqs amount:27670

seq avg len:27796.4344

total:77.38 Mb

depth:16.64X

As depicted in the following flowchart, AHA [6], Cerulean [7] and SSPACE-LongRead [8] are scaffolders that are able to use long reads (*e.g.* SMRT2) for scaffolding pre-assembled contigs (*e.g.* Abyss-assembled contigs). We used Abyss to assemble the short reads produce by MiSeq and then performed various scaffolders along with the PacBio long reads (*i.e.*, the filtered subreads). In addition, the long reads were corrected by LSC and PBcR pipeline using the short reads, also corrected by ECTools using the Abyss-assembled unitigs to produce corrected PacBio long reads (CPBLRs). We applied Patch to upgrade the draft assembly generated by Abyss to a hybrid assembly of high contiguous and accuracy. The QUAST-evaluated assembly results are shown in S4 Table. The commands we used are shown below:

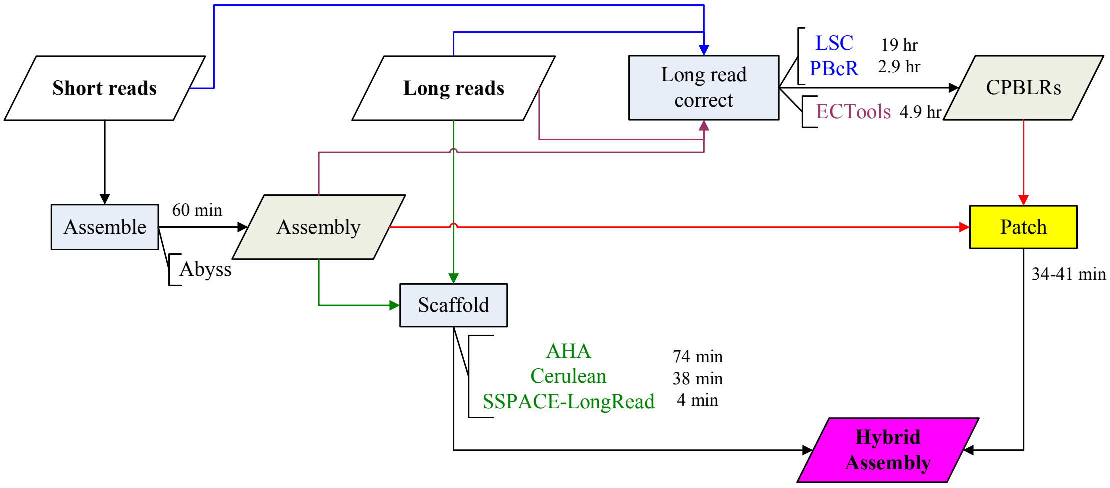

AHA

input.xml

<?xml version="1.0"?>

<pacbioAnalysisInputs>

<dataReferences>

<!-- High-confidence sequences fasta file -->

<url ref=" Abyss-contigs.fa "/>

<!-- PacBio reads, either in fasta or in bas.h5 format. -->

```
        <url ref="subreads.fasta"/>
        </dataReferences>
</pacbioAnalysisInputs>

source /opt/smrtanalysis/etc/setup.sh
smrtpipe.py --params=AHA.xml xml:input.xml
```

Cerulean 0.1.1

```
sawriter Abyss-contigs.fa
blasr subreads.fasta Abyss-contig.fa -minMatch 10 -minPctIdentity 70 -bestn 30 -nCandidates 30 -maxScore -500 -nproc 10 -noSplitSubreads -out mapping.fasta.m4
Cerulean.py --dataname Abyss --basedir for_cerulean --nproc 10
```

PBJelly.xml ##PBJelly (version 14.1.14)

```
<jellyProtocol>
    <reference> Abyss_cerulean.fasta</reference>
    <outputDir>Run_PBJelly</outputDir>
    <blasr>-minMatch 8 -minPctIdentity 70 -bestn 5 -nCandidates 20 -maxScore -500 -nproc 4 -noSplitSubreads</blasr>
    <input baseDir="Run_PBJelly/">
        <job>subreads.fastq</job>
    </input>
</jellyProtocol>

Jelly.py setup PBJelly.xml
Jelly.py mapping PBJelly.xml
Jelly.py support PBJelly.xml
Jelly.py extraction PBJelly.xml
Jelly.py assembly PBJelly.xml -x "--nproc=6"
Jelly.py output PBJelly.xml
```

SSPACE-LongRead 1.1

```
perl SSPACE-LongRead.pl -c Abyss-contigs.fa -p subreads.fastq -b Output
```

Unlike the above-mentioned scaffolders, Patch takes corrected long reads (CPBRLs) to improve pre-assembled contigs.

Patch

patch.config

```
source=/patch
in_ref=/ Abyss-contigs.fa
in_clr=/CPBLR.fasta
nucmer=/nucmer
makeblastdb=/makeblastdb
blastn=/blastn
2bwt-builder=/2bwt-builder
soap=/soap
read1=/MiSeq_Ecoli_MG1655_110721_PF_R1.fastq
read2=/MiSeq_Ecoli_MG1655_110721_PF_R2.fastq
min_i=262
max_i=333
genomesize=4650000

patch.py patch.config
```

We have tried to scaffold the Abyss-assembled contigs by SSPACE-LongRead using the corrected long reads (CPBLR1a), but only got a similar result to using SMRT1 (see S4 Table)

SSPACE-LongRead 1.1

```
perl SSPACE-LongRead.pl -c Abyss-contigs.fa -p CPBLR.fasta -b Output
```

Furthermore, because AHA and SSPACE-LongRead are able to take any pre-assembled assembly as an input for scaffolding using long reads, we used SPAdes 2.5.0 [9] to pre-assemble the short reads. Additionally, recent integration of Illumina short and PacBio long reads was implemented in SPAdes 3.0 [10], we conducted SPAdes 3.0 to produce hybrid assemblies for *E. coli*. For the sake of simplicity, we used the identical CPBLRs (*i.e.*, CPBLR1a-CPBLR2b) to upgrade the SPAdes-assembled contigs.

SPAdes's assembly [SPAdes 2.5]

```
spades.py -1 MiSeq_Ecoli_MG1655_110721_PF_R1.fastq -2
MiSeq_Ecoli_MG1655_110721_PF_R2.fastq -o Output
```

SPAdes's hybrid assembly [SPAdes 3.0]

```
spades.py -1 MiSeq_Ecoli_MG1655_110721_PF_R1.fastq -2
```

MiSeq_Ecoli_MG1655_110721_PF_R2.fastq --pacbio subreads.fastq -o Output

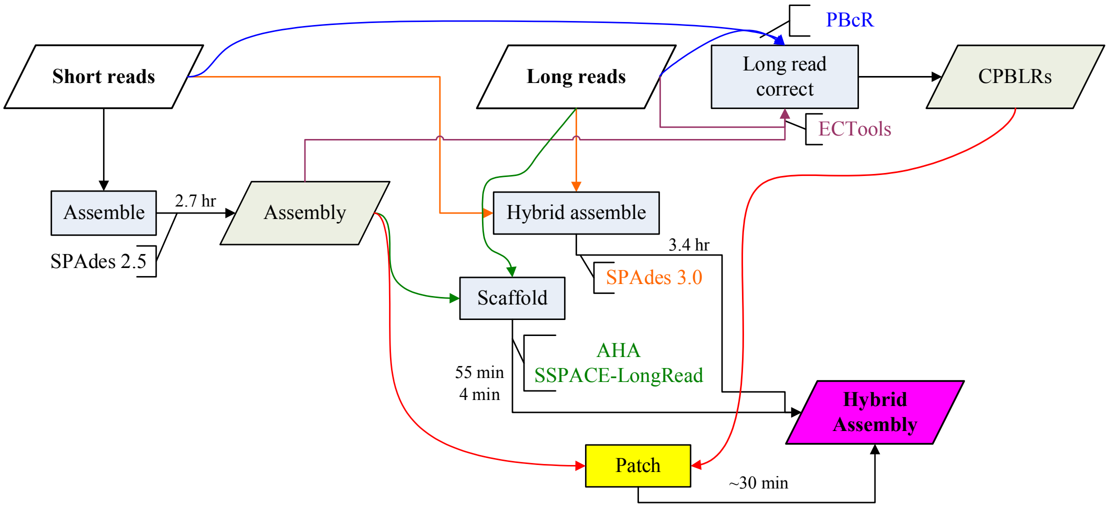

In addition to concatenating the assemblies generated from a single assembler (Abyss or SPAdes), we applied Patch to the assemblies obtained from the hybrid method: Celera Assembler [11].

Please note that we have encountered the following error messages when the version of PBcR pipeline, 8.2 beta, was performed on the subreads of SMRT1 and SMRT 2, respectively. We therefore conducted Celera Assembler to assemble the corrected long reads via runCA directly.

"Error: after correction only 6.40719032258064X for genome 4650000. Not performing automated assembly"

"Error: after correction only 9.45052387096774X for genome 4650000. Not performing automated assembly"

Celera Assembler:

runCA -p asm -d asm -s asm.spec PacBio_Illumina.frg

Patch without splitting

patch.config

source=/patch

in_ref=/assembly.fa

in_clr=/CPBLR.fasta

nucmer=/nucmer

makeblastdb=/makeblastdb

blastn=/blastn

patch.py patch.config

The QUAST-evaluated assembly results are shown in S4 Table

For *Meiothermus ruber* DSM1279:

We have downloaded the 454 sequencing reads from the Sequence Read Archive (SRR017780), and the PacBio long read of single SMRT cell (m120803_041200) from http://files.pacb.com/software/hgap/index.html. The filtered subreads were produced by running smrtpipe.py (SMRT analysis) with the following params.xml.

```
<param name="minLength">
  <value>50</value>
</param>
<param name="readScore">
  <value>0.75</value>
</param>
<param name="minSubReadLength">
  <value>50< /value>
```

Statistics of the filtered subreads (mruber.fastq):

seqs amount: 36180

seq avg len:2490.721448

total:90.11 Mb

depth: 29.07X

CPBLRs: The filtered subreads were corrected to long reads (CPBLRs) via invoking the PBcR command (8.2 beta) along with the 454 data:

```
fastqToCA -libraryname JR -technology 454 -reads JR.fastq > JR.frg
PBcR -length 500 -partitions 200 -l Pacbio_JR -s pacbio.spec -fastq mruber.fastq JR.frg
```

[28 min]

Statistics of CPBLRs:

seqs amount: 32083

seq avg len: 2076.73

total: 66.63 Mb

depth:21.49X

Besides, we have assembled the short reads with Newbler.

```
newAssembly 'Project'
addRun 'Project' 'SRR017780.sff'
runProject 'Project'
```

[10 min]

CPBLRs by ECTools: The filtered subreads were corrected to long reads (CPBLRs) by ECTools using the Newbler-assembled contigs:

Statistics of CPBLRs:

seqs amount: 29282

seq avg len: 2502.7752

total: 73.29 Mb

depth:23.64X

Patch without splitting

patch.config

source=/patch

in_ref=/454LargeContigs.fna

in_clr=/PacBio_JR.fasta

nucmer=/nucmer

makeblastdb=/makeblastdb

blastn=/blastn

patch.py patch.config

For *Pedobacter heparinus* DSM2366:

We have downloaded the Illumina Miseq sequencing reads from the Sequence Read Archive (SRR812176), and the PacBio long read of single SMRT cell (m120803_023226) from http://files.pacb.com/software/hgap/index.html. The filtered subreads were produced by running smrtpipe.py (SMRT analysis).

Statistics of the filtered subreads (phep.fastq):

seqs amount: 33630

seq avg len: 2493.151561

total: 83.84 Mb

depth: 16.22X

CPBLRs: The filtered subreads were corrected to long reads (CPBLRs) via invoking the PBcR command (8.2 beta) along with the Miseq data:

```
fastqToCA -libraryname Miseq -insertsize 256 59 -mates   SRR812176_1.fq, SRR812176_2.fq > Miseq.frg
PBcR -length 500 -partitions 200 -l Pacbio_Illumina -s pacbio.spec -fastq phep.fastq Miseq.frg
```

[1363 min]

Statistics of CPBLRs:

seqs amount: 32775

seq avg len: 2105.444119

total: 69.01 Mb

depth:13.35X

Besides, we have assembled the short reads with Abyss.

```
abyss-pe k=143 name=Abyss in='SRR812176_1.fq   SRR812176_2.fq'
```

[90 min]

Patch without splitting

patch.config

```
source=/patch
in_ref= /Contigs.fasta
in_clr=/PacBio.fasta
nucmer=/nucmer
makeblastdb=/makeblastdb
blastn=/blastn

patch.py patch.config
```

SPAdes 3.0 was used in this dataset, but got the unsatisfied assembly:

spades.py --12 SRR812176.fastq --pacbio phep.fastq -o output [673min]

**Statistics without reference**	**contigs**	**scaffolds**
# contigs	113	113
Largest contig	1 809 921	1 809 921
Total length	5 310 729	5 310 729
N50	1 265 182	1 265 182
**Misassemblies**		
# misassemblies	0	0
Misassembled contigs length	0	0
**Mismatches**		
# mismatches per 100 kbp	5.34	5.34
# indels per 100 kbp	0.43	0.43
# N's per 100 kbp	0	0
**Genome statistics**		
Genome fraction (%)	99.62	99.62
Duplication ratio	1.001	1.001
# genes	4324 + 6 part	4324 + 6 part
NGA50	1 265 182	1 265 182

In addition to the three bacterial species, we have applied Patch to assemble *S. cerevisiae* W303 genome. The short and long reads were downloaded from http://schatzlab.cshl.edu/data/ectools/ [12]. The short reads were assembled by Abyss:

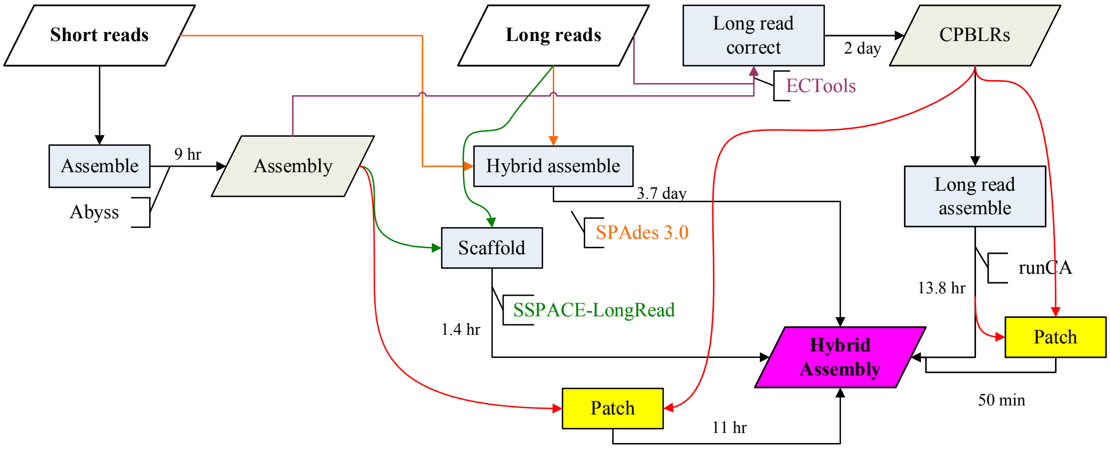

Abyss:

abyss-pe k=256 name=Abyss in= 'Illumina_500bp_2x300_R1.fastq Illumina_500bp_2x300_R2.fastq'   [541 min]

Sequences of the sixteen SMRT cells produced from PacBio RS II system for S. cerevisiae (yeast) were available in the website. We have downloaded the PacBio raw reads in fasta format and extracted sequence reads that belong to a single SMRT cell (m131225_191238_42137). Subsequently, we scaffolded the Abyss-assembled contigs with the long reads by SSPACE-LongRead and corrected the long reads to CPBLRs by ECTools. SPAdes was used to hybrid assemble the short and long reads for yeast genome. Please note that we performed all analysis on a server (Intel Xeon E7-4820, 2.00GHz with 256 GB of RAM). However, SPAdes crashed on this server, the assembly was thus computed on another sever with 512 GB of RAM. In addition, to utilize the CPBLRs by Patch, those sequences were *de novo* assembled by runCA. We also performed Patch to improve the runCA-assembled contigs.

Statistics of the filtered subreads (m131225_191238_42137.fa):

seqs amount: 44116

seq avg len: 5346.369231

total: 235.73 Mb

depth: 19.64X

SSPACE-LongRead 1.1

perl SSPACE-LongRead.pl -c Abyss-contigs.fa -p m131225_191238_42137.fa [81 min]

SPAdes's hybrid assembly [SPAdes 3.0]

spades.py -1 Illumina_500bp_2x300_R1.fastq -2 Illumina_500bp_2x300_R2.fastq --pacbio m131225_191238_42137.fa -o output [5404 min, using a server with 512 GB of RAM]

Statistics of CPBLRs (corrected.long.fa):

seqs amount: 21112

seq avg len: 7072.946997

total: 149.32 Mb

depth:12.44X

Patch (Abyss + Patch)

patch.config

source=/patch

in_ref=/Abyss.ctg.fa

in_clr=/corrected.long.fa

nucmer=/nucmer

makeblastdb=/makeblastdb

blastn=/blastn

2bwt-builder=/2bwt-builder

soap=s/oap

read1=/Illumina_500bp_2x300_R1.fastq

read2=/Illumina_500bp_2x300_R2.fastq

min_i=400

max_i=600

patch.py patch.config

Celera Assembler:

fastaToCA -l CorrectedLongRead -s corrected.long.fa -q ec.qual > my.frg

runCA ovlMinLen=100 ovlErrorRate=0.02 utgGraphErrorRate=0.01 utgGenomeSize=12000000 unitigger=bogart -p asm -d asm my.frg [825 min]

Patch without splitting (runCA + Patch)

patch.config

source=/patch

in_ref=/asm.ctg.fasta

in_clr=/corrected.long.fa

nucmer=/nucmer

makeblastdb=/makeblastdb

blastn=/blastn

patch.py patch.config

References:

1. Koren S, Harhay GP, Smith TP, Bono JL, Harhay DM, McVey SD, Radune D, Bergman NH, Phillippy AM: **Reducing assembly complexity of microbial genomes with single-molecule sequencing**. *Genome Biol* 2013, **14**(9):R101.
2. Simpson JT, Wong K, Jackman SD, Schein JE, Jones SJM, Birol I: **ABySS: A parallel assembler for short read sequence data**. *Genome Research* 2009, **19**(6):1117-1123.
3. Au KF, Underwood JG, Lee L, Wong WH: **Improving PacBio long read accuracy by short read alignment**. *PLoS One* 2012, **7**(10):e46679.
4. Salmela L, Rivals E: **LoRDEC: accurate and efficient long read error correction**. *Bioinformatics* 2014, **30**(24):3506-3514.
5. Hackl T, Hedrich R, Schultz J, Forster F: **proovread: large-scale high-accuracy PacBio correction through iterative short read consensus**. *Bioinformatics* 2014, **30**(21):3004-3011.
6. Bashir A, Klammer AA, Robins WP, Chin CS, Webster D, Paxinos E, Hsu D, Ashby M, Wang S, Peluso P *et al*: **A hybrid approach for the automated finishing of bacterial genomes**. *Nature biotechnology* 2012.
7. Deshpande V, Fung ED, Pham S, Bafna V: **Cerulean: A hybrid assembly using high throughput short and long reads**. In: *Algorithms in Bioinformatics.* Springer; 2013: 349-363.
8. Boetzer M, Pirovano W: **SSPACE-LongRead: scaffolding bacterial draft genomes using long read sequence information**. *BMC Bioinformatics* 2014, **15**:211.
9. Bankevich A, Nurk S, Antipov D, Gurevich AA, Dvorkin M, Kulikov AS, Lesin VM, Nikolenko SI, Pham S, Prjibelski AD *et al*: **SPAdes: a new genome assembly algorithm and its applications to single-cell sequencing**. *Journal of computational biology : a journal of computational molecular cell biology* 2012, **19**(5):455-477.
10. Prjibelski AD, Vasilinetc I, Bankevich A, Gurevich A, Krivosheeva T, Nurk S, Pham S, Korobeynikov A, Lapidus A, Pevzner PA: **ExSPAnder: a universal repeat resolver for DNA fragment assembly**. *Bioinformatics* 2014, **30**(12):i293-i301.
11. Koren S, Schatz MC, Walenz BP, Martin J, Howard JT, Ganapathy G, Wang Z, Rasko DA, McCombie WR, Jarvis ED *et al*: **Hybrid error correction and de novo assembly of single-molecule sequencing reads**. *Nature biotechnology* 2012.
12. Lee H, Gurtowski J, Yoo S, Marcus S, McCombie WR, Schatz M: **Error correction and assembly complexity of single molecule sequencing reads**.

BioRxiv 2014.
