# Supplementary material for: Evaluation and Validation of Assembling Corrected PacBio Long Reads for Microbial Genome Completion via Hybrid Approaches
Source: PLoS One. 2015 Dec 7;10(12):e0144305. doi: 10.1371/journal.pone.0144305 (PMC4671558; doi:10.1371/journal.pone.0144305)

S2 Appendix

Here we provide more details about the algorithms of Patch. We take Abyss-assembled contigs, corrected PacBio long reads (CPBLR1a, SMRT1 corrected by PBcR pipeline) and Miseq short reads as inputs to Patch.

myconfig:

```
source=/ patch
in_ref=/Abyss.ctg.fa
in_clr=/Pacbio_Illumina.fasta    # CPBLR1a were generated by correcting the subreads of SMRT1 data by PBcR pipeline with Miseq short reads
nucmer=/nucmer
makeblastdb=/makeblastdb
blastn=/blastn
2bwt-builder=/2bwt-builder
soap=/soap
read1=/MiSeq_Ecoli_MG1655_110721_PF_R1.fastq
read2=/MiSeq_Ecoli_MG1655_110721_PF_R2.fastq
min_i=262
max_i=333
genomesize=4650000
```

```
$ python patch.py myconfig
whole:4647119
N50: 656546
Number of contigs: 10
```

Length of the longest contig: 1808593

Step 1: Assembly modification

Input: my_ref.fa and my_CLR.fa

If the genome size was specified in the config file, the longest 15X CPBLRs are selected and saved as my_CLR.fa.Long.fa

---Gap-Filling---

Inupt: my_ref.fa and my_CLR.fa.Long.fa

Any N within a contig defines a gap. The 100-bp flanking sequences of the gap are used to identify those CPBLRs spanning the gap. We replace the gap with the corresponding sequence which has multiple and maximal occurrences. The schematic diagram for gap-filling is shown in Fig. 1A. As can be seen in **Filled_info.txt**, there were 9 gaps in the Abyss-assembled contigs and the nine gaps were filled by Patch using the corresponding sequences of CPBLRs. After gap-filling, Filled_my_ref.fa is produced.

```
>ref_66_len:64344
clr:>clr_8229_len:5928 gap_related_seq:CTCCCGCCGTACCTGTT
clr:>clr_1547_len:6027 gap_related_seq:CTCCCGCCGTACCTGTT
clr:>clr_2029_len:5468 gap_related_seq:CTCCCGCCGTACCTGTT
Replace with :CTCCCGCCGTACCTGTT
>ref_133_len:66658
clr:>clr_9785_len:2199 gap_related_seq:AAACCGCTGGATAAAACCCGCGCTAATTCCCT
clr:>clr_7310_len:1869 gap_related_seq:AAACCGCTGGATAAAACCCGCGCTAATTCCCT
clr:>clr_5549_len:6242
gap_related_seq:AAACCGCTGGATAAAACCCGCGCTAATTCCCTGACCCGCCACTTTATCACTCAGCATCTACCCACCGATTAACCGAG
GAGACGTGATGTCCCGAGCGGCGCTGGGGCACCAACCGCTAGAAAAACACCCGCGCTAATTCCCT
clr:>clr_7939_len:4000 gap_related_seq:AAACCGCTGGATAAAACCCGCGCTAATTCCCT
```

Replace with :AAACCGCTGGATAAAACCCGCGCTAATTCCCT
...

---Splitting---
Input: Filled_my_ref.fa and short reads
We have used SOAP2 (SOAP aligner) to align short reads to the Filled_my_ref.fa so as to estimate read coverage statistics. Contigs are split or trimmed (the 1% end-regions of contigs) in the zero-coverage positions (as shown in Fig. 1B). Contigs larger than 50 bp are saved as **Split_Ref.fa**.

For example,
ref_3_len:35361 was trimmed to ref_3_35361_trimmed_1_len:35359
ref_133_len:66658 was split into ref_133_66656_split_1_len:12196, ref_133_66656_split_2_len:29616,and ref_133_66656_split_3_len:24699

Step 2: Representative sequence selection
---Contig/CPBLR selection---
Input: Split_Ref.fa or Filled_my_ref.fa and my_CLR.fa.Long.fa in Step 1
We concatenate **Split_Ref.fa** (or **Filled_my_ref.fa**) and **my_CLR.fa.Long.fa** into a single file. We then employ NUCmer with the default setting to perform sequence alignment (one vs. all-minus-one). We select the longest sequence among contigs and CPBLRs as a representative sequence and remove the contigs and CPBLRs whose major portion of sequence (>95% alignment rate and sequence identity) is found in the representative sequence. The longest sequence of the remaining sequences is iteratively selected as a subsequent representative sequence until no sequence remained. The IDs of the removed sequences can be found in **explained.txt.** The representative contigs and CPBLRs are stored in the file of **Merge_Contigs.fa**.
For example, as data shown in the file of explained.txt:

RefID: ref_50_203438_trimmed_1_len:203435

clr_3130_len:4055 clr_7724_len:1032 clr_8616_len:767 clr_8015_len:931......(over 380 IDs)

We firstly selected the longest sequence ref_50_203438_trimmed_1_len:203435 as a representative sequence and performed sequence alignment for getting the explained information so as to remove the explained sequences (i.e., clr_3130_len:4055 clr_7724_len:1032 clr_8616_len:767 clr_8015_len:931....). The second longest sequence ref_77_222518_split_2_len:194038 was selected as a representative sequence and performed sequence alignment for getting the explained information and so on and so forth.

Step3: Iterative connection

Input: Merge_Contigs.fa in step 2

---Strict bridging---

The representative sequences were self-aligned with blastn (all vs. all) to identify end-to-end overlaps. The blast results are saved in **My_result.txt**. If a representative sequence uniquely bridged either two representative contigs or one representative contig and one CPBLR, the representative sequence was selected as a candidate for connection. Here, we defined "strict bridging" for the unique bridge whose ends were aligned to a single end of the sequence of choice, i.e. the length of end-to-end alignment was maximal and at least 1.25-fold larger than the secondary choice.

To demonstrate "strict bridging", we selected two CPBLRs as examples:

BLASTN 2.2.25+

Query: clr_72_len:9111

Database: Side_DB

Fields: query id, subject id, q. start, q. end, s. start, s. end, alignment length, evalue, score

6 hits found

clr_72_len:9111 clr_72_len:9111 1 9111 1 9111 9111 0.0 9111

clr_72_len:9111 ref_3_35361_trimmed_1_len:35359 2 6622 6621 1 6621 0.0 6621

clr_72_len:9111 ref_79_115397_split_4_len:71291 6616 9111 71291 68796 2496 0.0 2496

clr_72_len:9111 clr_653_len:6606 3551 3603 73 21 53 4e-13 41

clr_72_len:9111 clr_141_len:8437 3551 3603 1124 1176 53 4e-13 41

clr_72_len:9111 ref_31_8924_trimmed_1_len:8921 3551 3603 4837 4785 53 4e-13 41

We selected clr_72_len:9111 as a candidate for connection between ref_3_35361_trimmed_1_len:35359 and ref_79_115397_split_4_len:71291, because its ends are uniquely aligned to these two sequence ends, respectively. Similar bridging candidates are listed in **Filtered_1_Bridges_clr_as_Bridge.txt**.

One the contrary, the following CPBLR (clr_194_len:8030) was not selected for strict bridging because we request that the length of end-to-end alignment is maximal and at least 1.25-fold larger than the secondary choice.

BLASTN 2.2.25+

Query: clr_194_len:8030

Database: Side_DB

Fields: query id, subject id, q. start, q. end, s. start, s. end, alignment length, evalue, score

31 hits found

clr_194_len:8030 clr_194_len:8030 1 8030 1 8030 8030 0.0 8030

clr_194_len:8030 ref_24_179382_split_1_len:45827 1 7610 7610 1 7610 0.0 7607 # the maximal front-end alignment

clr_194_len:8030 clr_361_len:7395 577 5248 1 4672 4672 0.0 4672

clr_194_len:8030 clr_1350_len:5451 3359 8030 2 4673 4672 0.0 4669 # the maximal back-end alignment

clr_194_len:8030 clr_811_len:6257 3655 8030 1 4376 4376 0.0 4373 # the second back-end alignment, 4669/4373<1.25

clr_194_len:8030 clr_828_len:6229 5877 8030 6229 4076 2154 0.0 2151

......

Although the front end of clr_194_len:8030 uniquely aligns to ref_24_179382_split_1_len:45827, its back end ambiguously aligns to multiple sequences. Such a sequence was not selected for connection and its blast results are listed in **Bad_Bridge.txt**.

The candidate CPBLRs were ordered by length from longest to shortest and bridged two adjacent sequences (**Filtered_1_Bridges_clr_as_Bridge.txt**). The connection information are saved in **2_aliases.txt**. Please note that we connect two representative contigs prior to connect one representative contig with one representative CPBLR.

If the two adjacent sequences bridged by a CPBLR overlapped, the overlapping sequence of the shorter segment was trimmed to connect with the other sequence. If a CPBLR spanned a gap between two adjacent sequences, the gap was filled with the corresponding sequence of CPBLR. For examples,

Overlapped sequences were connected by a CPBLR:

Connected_Contig_1_1 from: ref_3_35361_trimmed_1_len:35359 clr_72_len:9111 ref_79_115397_split_4_len:71291 [see **2_aliases.txt**]

In **Filtered_1_Bridges_clr_as_Bridge.txt**

clr_72_len:9111 ref_3_35361_trimmed_1_len:35359 2 6622 6621 1 6621 0.0 6621

clr_72_len:9111 ref_79_115397_split_4_len:71291 6616 9111 71291 68796 2496 0.0 2496

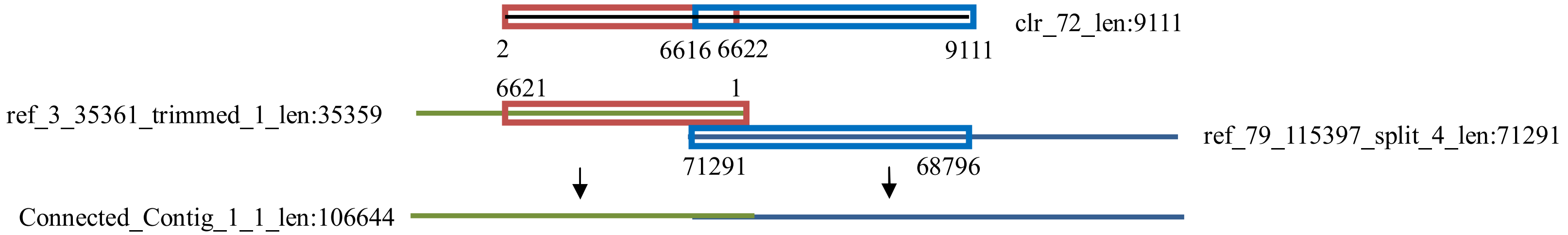

Therefore, Patch produced Connected_Contig_1_1_len:106644 in 2_Contigs_B.fa.

A sequence gap between two adjacent sequences was filled by a CPBLR:

Connected_Contig_1_2 from: ref_120_133699_trimmed_1_len:133696 clr_98_len:8801 ref_4_55928_trimmed_1_len:55924 [see **2_aliases.txt**]

In **Filtered_1_Bridges_clr_as_Bridge.txt**

clr_98_len:8801 ref_120_133699_trimmed_1_len:133696 1 3677 3677 1 3677 0.0 3674

clr_98_len:8801 ref_4_55928_trimmed_1_len:55924 3706 8801 55924 50829 5096 0.0 5096

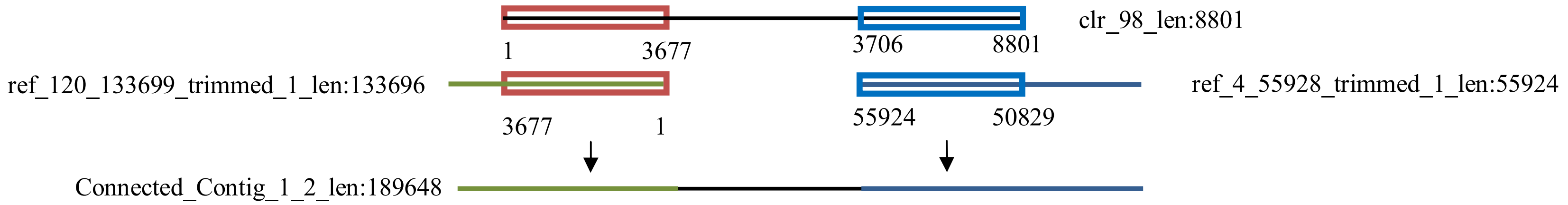

Therefore, Patch produced Connected_Contig_1_2_len:189648 in **2_Contigs_B.fa**.

This process (strict bridging) was performed iteratively until no CPBLR remained for bridging. We then employed the connected sequences as candidates for connection and performed strict bridging iteratively. As can be seen in the fold of strict_bridging_5, no CPBLR can be used for bridging, we therefore take contigs as candidates for connection (**Filtered_1_Bridges_assembler_ctg_as_Bridge.txt**).

---Easy bridging---

At the end of strict bridging, we prepared a new list of CPBLR for "easy bridging". If a CPBLR are end-to-end overlapping with only two individual contig sequences, we keep this CPBLR. Otherwise we remove such a CPBLR and get its explaining sequences, if any, based on the file of **explained.txt** in Step 2.

For examples, as shown in Bad_Bridge.txt (under the fold of strict_bridging_8)

clr_468_len:7059 clr_534_len:6882 1 5840 6020 182 5840 0.0 5836

clr_468_len:7059 Connected_Contig_7_1_len:972994 1 5383 967612 972994 5383 0.0 5383

clr_468_len:7059 ref_82_226124_split_1_len:10811 5498 7059 10811 9250 1562 0.0 1562
We found that clr_468_len:7059 can be used to bridge Connected_Contig_7_1_len:972994 and ref_82_226124_split_1_len:10811 without considering the interference of clr_534_len:6882, such a CPBLR was therefore kept for the following analysis (easy bridging).

On the contrary, we found the front and back ends of clr_916_len:6071 ambiguously align to multiple sequences and would like to remove this CPBLR. We traced back to the file of **explained.txt** in Step 2 and got its explaining CPBLRs (e.g., clr_4803_len:2618 and clr_6558_len:1494) back.
clr_916_len:6071 ref_72_8072_trimmed_1_len:8047 1 1735 6313 8047 1735 0.0 1735
clr_916_len:6071 Connected_Contig_6_1_len:294491 1 777 293588 294364 777 0.0 777
clr_916_len:6071 Connected_Contig_3_2_len:52537 3951 6071 52537 50417 2121 0.0 2121
clr_916_len:6071 Connected_Contig_5_3_len:213812 4936 6071 213812 212677 1136 0.0 1136
clr_916_len:6071 Connected_Contig_5_15_len:69896 5203 6071 1 869 869 0.0 869
clr_916_len:6071 Connected_Contig_5_12_len:169026 5839 6071 1 233 233 2e-118 230

We therefore produced a file named **Connected_Result_1.fa** in the fold of easy_bridging_A.
Prior to perform easy bridge, the sequences of **Connected_Result_1.fa** were self-aligned by blastn to estimate the maximal size of the repetitive regions. Possible repetitive regions were stored in **Repeat_Region.txt**, and the maximal size was estimated (2879) and recorded in **RR.txt**.

We examined each of the CPBLRs in **Connected_Result_1.fa** to determine whether they were capable of bridging two sequences (not bridging CPBLRs), *i.e.* both ends of a CPBLR are end-to-end overlapping with only two individual contig sequences. Such a CPBLR was selected as a candidate for easy bridging if its length was greater than the maximum size of the repetitive regions. The candidate CPBLRs were ordered by length from longest to shortest and bridged two adjacent sequences (**keep_clr_N.txt**), and the connection information are saved in **aliases_N.txt**. The process of easy bridging was also performed iteratively.

---Overlapping---

After the strict and easy bridging processes, the connected sequences were self-aligned by blastn. If two sequences comprised a proper end-to-end overlap and the overlapping length was greater than the maximum size of the repetitive regions, they were connected. We have analyzed the end-to-end overlapping conditions and divided into **Extend_h.txt** and **Extend_t.txt**. The overlapping information are saved in **Extend_info**.

For example,

Connected_Contig_5_7_len:121174 Connected_Contig_5_15_len:69896 1 3095 66802 69896 3095 0.0 3095 [see **Extend_h.txt**]

A contig Overlap_1_1_len:187975 was therefore produced by connecting the overlapping sequences Connected_Contig_5_7_len:121174 and Connected_Contig_5_15_len:69896. [see **Extend_info**]

---Easy bridging---

Finally, an alternative easy-bridging process was iteratively performed to ensure that each candidate CPBLR for bridging had been used.

In this case, no further CPBLR can be used in the process of easy_bridging_B. The whole process was ended when Connected_Results_N.fa was identical to Connected_Results_N-1.fa. A file named **patched.ctg.fa** was accordingly produced as the end product of Patch.

Through the whole process of Patch, the abyss-assembled contigs (left figure) were upgraded to the patched contigs (right figure). Obviously, most of the dark-grey segments (representing overlaps between contigs) can be eliminated by the support of spanning long reads, however, some of the gaps (white segments in the inner circle) cannot be filled because of repetitive sequences.

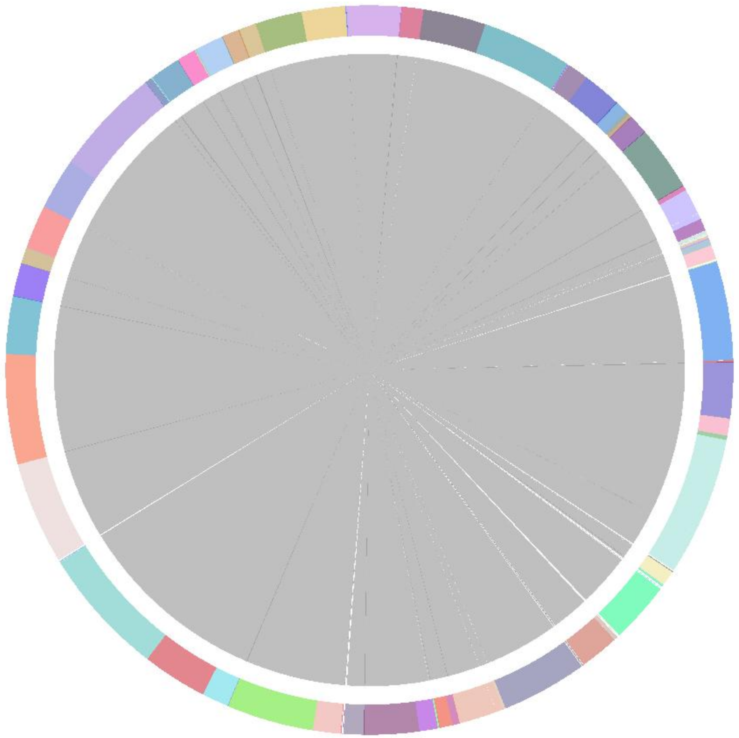

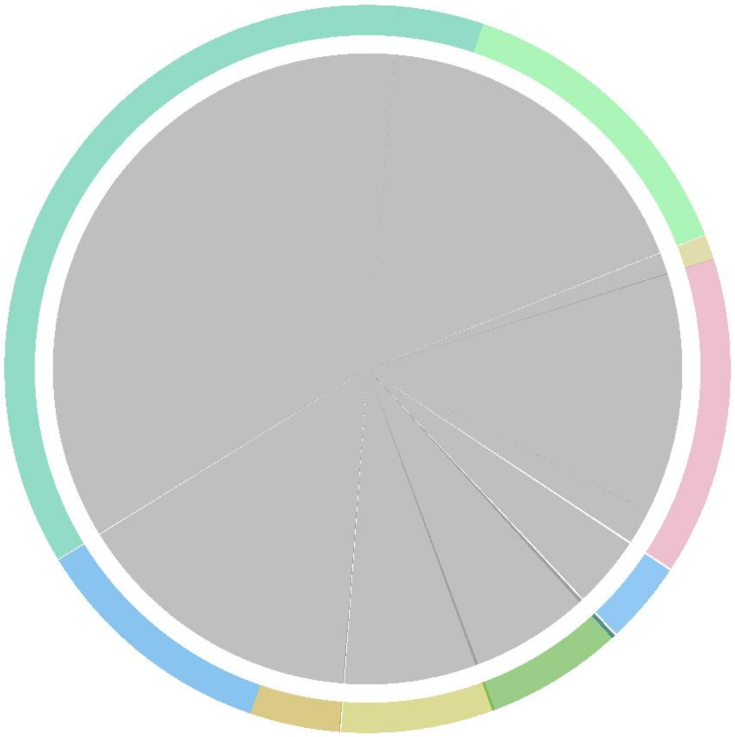

Supplement: S2 Appendix — (PDF) [file pone.0144305.s002.pdf]
