# Supplementary material for: Evaluation and Validation of Assembling Corrected PacBio Long Reads for Microbial Genome Completion via Hybrid Approaches
Source: PLoS One. 2015 Dec 7;10(12):e0144305. doi: 10.1371/journal.pone.0144305 (PMC4671558; doi:10.1371/journal.pone.0144305)
Supplement: S2 File — Sequence assembly produced by Patch for S. cerevisiae W303 (Figure A). Sequence assemblies produced by AHA (Figure B), SSPACE-LongRead (Figure C) and Cerulean (Figure D) for E. coli MG1655. (PDF) [file pone.0144305.s004.pdf]

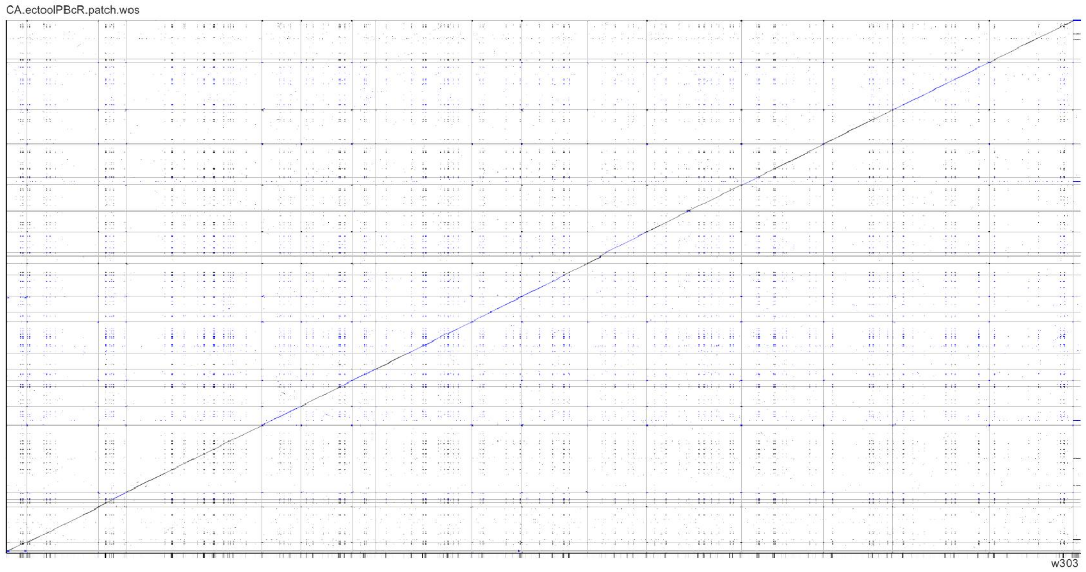

Figure A Dot plots of sequence assembly for *S. cerevisiae* W303. Assembly produced by Patch using runCA-assembled contigs and ECTools-corrected long reads as inputs. (Reference genome: 18 scaffolds from NCBI)

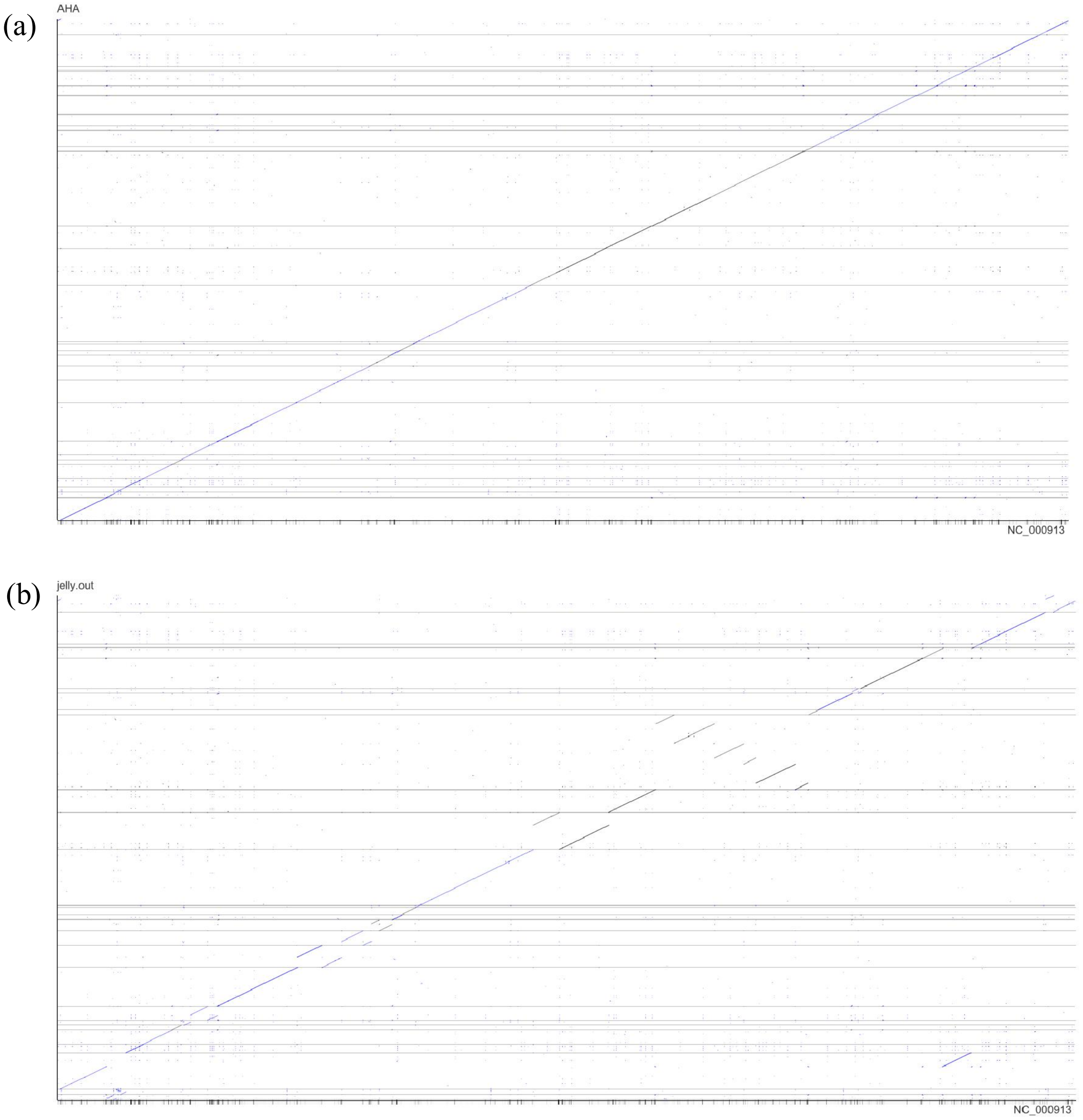

Figure B Dot plots of sequence assemblies produced by (a) AHA and (b) AHA followed by PBJelly using Abyss-assembled contigs and SMRT1 as inputs.

(a)

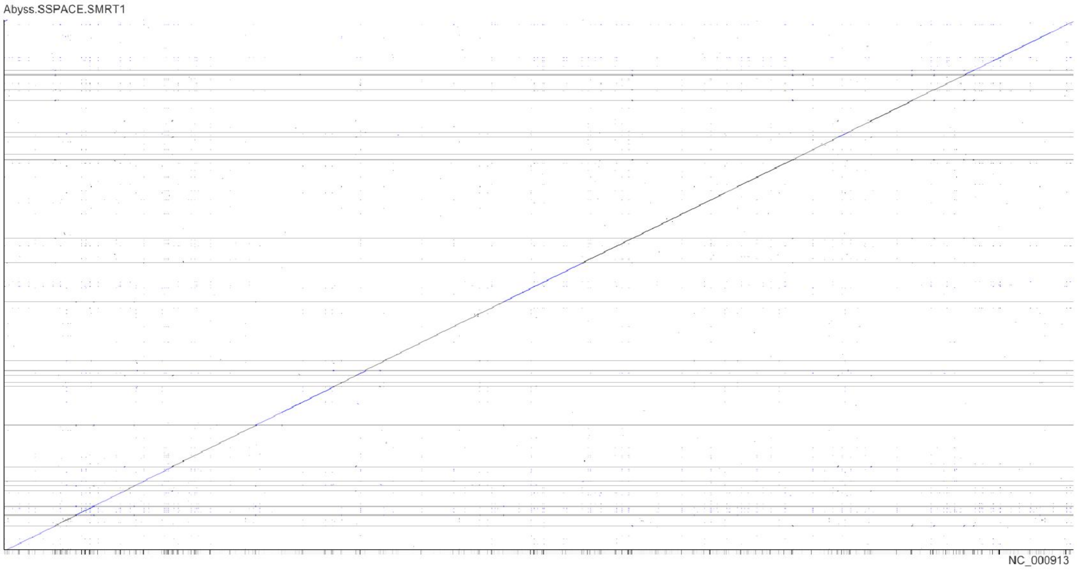

(b)

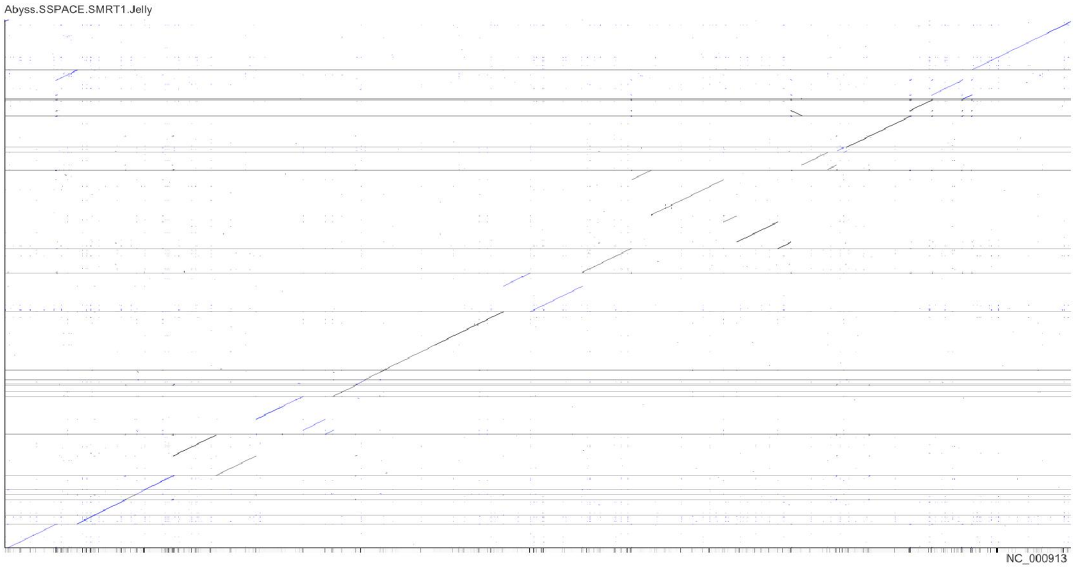

Figure C Dot plots of sequence assemblies produced by (a) SSPACE-LongRead and (b) SSPACE-LongRead followed by PBJelly using Abyss-assembled contigs and SMRT1 as inputs.

(a)

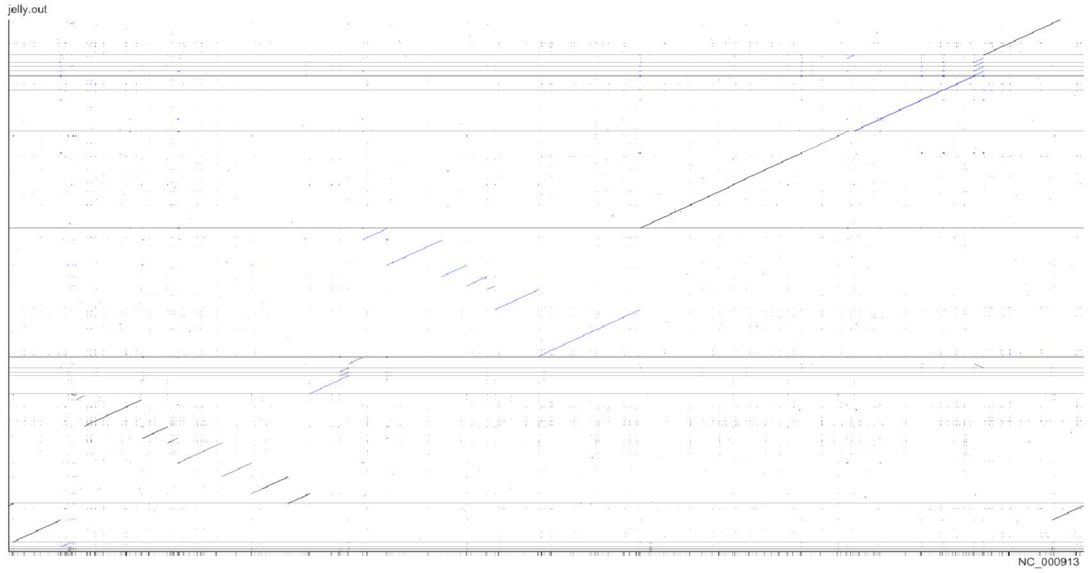

(b)

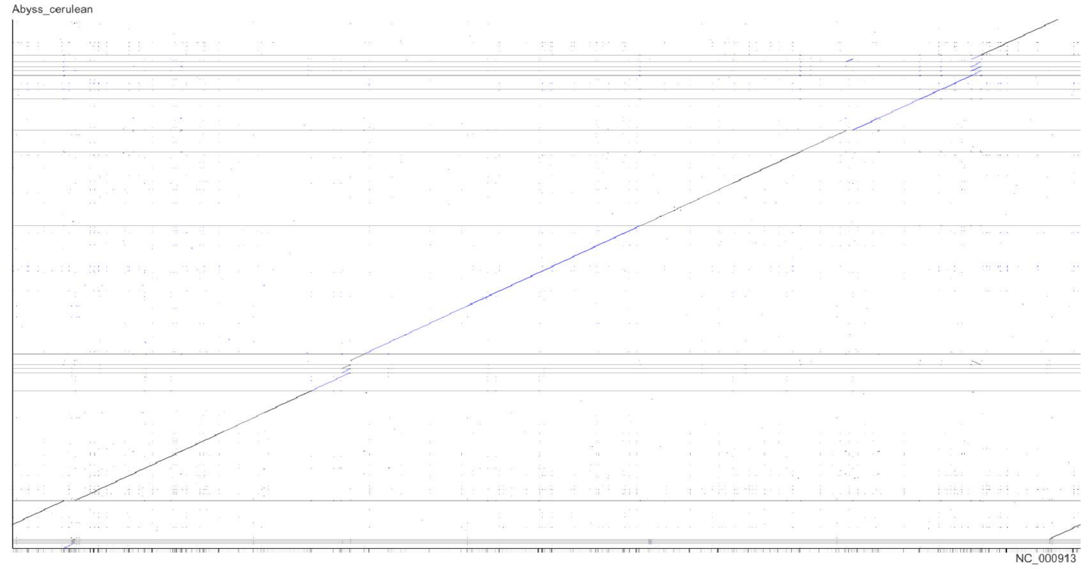

Figure D Dot plots of sequence assemblies produced by Cerulean (a) via post-process with PBJelly and (b) without PBJelly using Abyss-assembled contigs and SMRT1 as inputs.
